# Phase II trial of CDK4/6 inhibitor palbociclib in advanced sarcoma based on mRNA expression of *CDK4/CDKN2A*

**DOI:** 10.1038/s41392-023-01661-8

**Published:** 2023-10-25

**Authors:** Javier Martin-Broto, Jeronimo Martinez-Garcia, David S. Moura, Andres Redondo, Antonio Gutierrez, Antonio Lopez-Pousa, Javier Martinez-Trufero, Isabel Sevilla, Roberto Diaz-Beveridge, Maria Pilar Solis-Hernandez, Amancio Carnero, Marco Perez, David Marcilla, Jesus Garcia-Foncillas, Pablo Romero, Javier Fernandez-Jara, Daniel Lopez-Lopez, Ivan Arribas, Nadia Hindi

**Affiliations:** 1 Health Research Institute-Fundación Jiménez Díaz University Hospital, Universidad Autónoma de Madrid (IIS-FJD, UAM), 28040 Madrid, Spain; 2 Medical Oncology Department, Fundación Jimenez Diaz University Hospital, 28040 Madrid, Spain; 3 General de Villalba University Hospital, 28400 Madrid, Spain; 4 Medical Oncology Department, University Hospital Virgen de la Arrixaca, 30120 Murcia, Spain; 5 Department of Medical Oncology, Hospital Universitario La Paz-IdiPAZ, P. Castellana, 261, 28046 Madrid, Spain; 6 Hematology Department, University Hospital Son Espases, 07120 Mallorca, Spain; 7 Medical Oncology Department, Sant Pau Hospital, 08025 Barcelona, Spain; 8 Medical Oncology Department, University Hospital Miguel Servet, 50009 Zaragoza, Spain; 9 Investigación Clínica y Traslacional en Cáncer/ Instituto de Investigaciones Biomédicas de Malaga (IBIMA)/ Hospitales Universitarios Regional y Virgen de la Victoria de Malaga, Malaga, Spain; 10 Medical Oncology Department, Hospital Universitari i Politècnic La Fe, 46026 Valencia, Spain; 11 Medical Oncology Department, Central University Hospital of Asturias, 33011 Oviedo, Spain; 12 Instituto de Biomedicina de Sevilla (IBiS; HUVR, CSIC, US), 41013 Sevilla, Spain; 13 Pathology Department, Virgen del Rocio University Hospital, 41013 Sevilla, Spain; 14 Radiology Department, Fundación Jimenez Diaz University Hospital, 28040 Madrid, Spain; 15 Computational Medicine Platform, Fundación progreso y salud (FPS), Hospital Virgen del Rocío, 41013 Seville, Spain; 16 Bioinformatics in Rare Diseases (BiER). Centro de Investigación Biomédica en Red de Enfermedades Raras (CIBERER), FPS, Hospital Virgen del Rocio, Sevilla, Spain; 17 Universitat de València - ERI-CES, 46010 Valencia, Spain

**Keywords:** Sarcoma, Translational research, Predictive markers

## Abstract

Cyclin-dependent kinases 4 and 6 (*CDK4/6*) inhibitors demonstrated activity in terms of progression-free survival (PFS) in advanced dedifferentiated liposarcoma (DD-LPS), a sarcoma with CDK4 amplification. CDK4 overexpression is by far more common than amplification in sarcomas and it might be a rational target for CDK inhibitors. Preclinical investigators of this study found that *CDK4* overexpression, while not of *CDKN2A*, was the most consistent predictive factor for palbociclib efficacy in sarcomas. Advanced adult-type soft-tissue sarcoma, excluding DD-LPS, or bone sarcoma patients, progressing after at least one systemic line, whose tumors overexpressed *CDK4*, but not *CDKN2A* at baseline biopsy, were accrued in this single-arm phase II trial (EudraCT number: 2016-004039-19). With the main endpoint of a 6-month PFS rate, 40% was considered promising in this population. Palbociclib was administered orally at 125 mg/day for 21 days in 28-day cycles. A total of 214 patients with 236 *CDK4/CDKN2A* determinations were assessed for prescreening, archival material (141), and screening, baseline biopsy (95). There were 28 (29%) with favorable mRNA profiles from 95 screened patients at baseline. From 23 enrolled patients, 21 evaluable, the 6-month PFS rate was 29% (95% CI 9–48), and there were 6 patients out of 21 with a PFS longer than 6 months. The median PFS and overall survival were 4.2 (95% CI 3.6–4.8) and 12 (95% CI 8.7–15.4) months, respectively. Translational research showed a significant correlation between CDK4 mRNA and protein expression. Palbociclib was active in a variety of sarcoma subtypes, selected by *CDK4/CDKN2A,* and deserves further investigation in the sarcoma context.

## Introduction

Sarcomas constitute a heterogeneous family of malignant tumors, generally derived from mesoderm that exhibit a different behavior across distinct subtypes. Even though targeted therapies have emerged for some rare sarcomas such as alveolar soft part sarcoma,^1,2^ solitary fibrous tumor,^3,4^ PEComa,^5^ inflammatory myofibroblastic tumor,^6^ or myxoid extraskeletal chondrosarcoma,^7^ among others, the truth is that for the most frequent sarcoma subtypes, excluding GIST, chemotherapy is still the backbone for the first and second line of systemic treatment.

Patients with advanced sarcomas still represent a poor prognostic population, exhibiting a median of overall survival (OS) that has increased above 18 months in recent phase III trials for soft tissue sarcoma (STS) patients,^8,9^ and below one year in metastatic relapsed osteosarcoma with the exclusion of surgically rescued cases.^10–12^

Early cell-cycle control proteins, involved in the G1-S transition, such as D cyclins and cyclin-dependent kinases (CDK) 4 and 6, are frequently dysregulated in different sarcomas.^13^ Somatic copy number alterations are the characteristic genomic finding affecting the axis *CDKN2A-CDK4-RB1*. Specifically, *CDK4* amplifications and *CDKN2A* deep deletions were found in 86% and 2% of dedifferentiated liposarcomas (DD-LPS), respectively. While *CDKN2A* deep deletions were found in 8% of leiomyosarcomas (LMS), 20% of undifferentiated pleomorphic sarcomas (UPS) and 18% of myxofibrosarcomas.^14^ These findings constituted the basis of the exploration of palbociclib and abemaciclib, CDK4 inhibitors, in DD-LPS.^15,16^ Nonetheless, the proportion of STS patients with CDK4 amplification is lower than 5% apart from DD-LPS.^17^ The lack of a benign counterpart in the majority of sarcoma subtypes makes it more challenging to determine what might be considered categorically the threshold of mRNA overexpression. However, CDK4 overexpression is by far more common than amplification in sarcomas and it might be a rational target for CDK inhibitors. Yet, the general assumption that gene amplification causes overexpression is not universally accepted since convincing experimental corroboration is lacking for most amplified oncogenes. In fact, there are several examples of a lack of correlation between amplification and overexpression.^18^ Some studies have demonstrated that CDK4 was overexpressed in sarcomas and that this overexpression entailed a worse prognosis, and when it was downregulated, proliferation was inhibited.^19^ However, to date, no conclusive data have been derived from the plethora of potential predictive biomarkers of CDK4 inhibitory studies.

In preclinical studies, we found that the best predictive biomarker for palbociclib efficacy was *CDK4* mRNA overexpression while *CDKN2A* was not overexpressed, in 16 sarcoma models used for in vitro and in vivo experiments.^20^ This finding led us to explore palbociclib in a phase II trial, in a wide range of sarcoma subtypes, apart from DD-LPS, Ewing sarcoma, and rhabdomyosarcoma, overexpressing *CDK4* without overexpression of *CDKN2A*. As the exhibited key feature of CDK4/6 inhibition is the cell cycle inhibition, mimicking senescence biological phenotype, we expected an increase in disease control by extending the progression-free survival (PFS) rather than increasing the overall response rate (ORR).

## Results

**Fig. 1:**
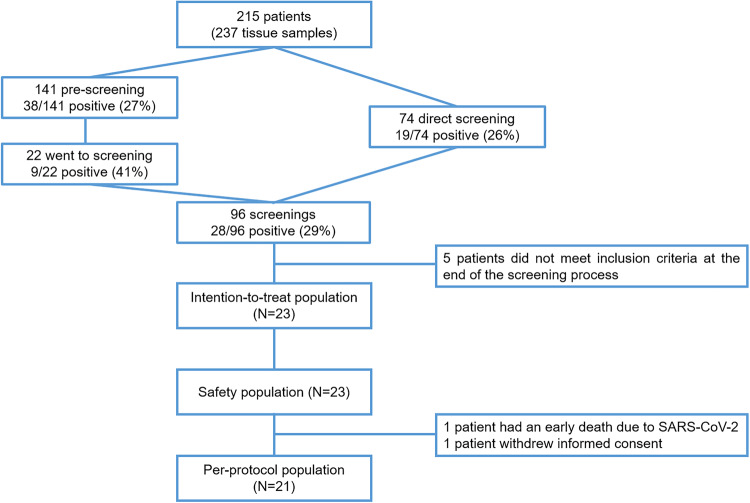
Consort diagram

From December 2018 to November 2021, 96 patients with advanced and progressing sarcomas were screened for *CDK4* and *CDKN2A* RNA expression in a baseline biopsy and assessed for eligibility (Fig. 1). There were 38 out of 141 (27%) and 28 out of 96 (29%) cases that fit with the pre-specified RNA expression from pre-screening (archival material) and screening (baseline biopsy) analysis, respectively. Besides, 9 out of 22 (41%) patients had both pre-screening and screening RNA expressions within the values of the protocol requirement. Eventually, 23 patients were accrued in 9 Spanish hospitals which resulted in 21 evaluable patients. One patient died after SARS-Cov2 infection in the first month of enrollment and another patient withdrew the informed consent 2 weeks after starting the treatment (Fig. 1).

The clinical cutoff for the final data analysis was August 29, 2022. At that time, 1 out of the 23 who had started the treatment (4%) was still receiving palbociclib and 22 (96%) had discontinued palbociclib. In addition to the early death mentioned before, 20 patients discontinued because of progression. 21 patients constituted the per-protocol population, and were consequently evaluable for efficacy, while 23 were evaluable for toxicity. A total of 120 one-month cycles were given to the enrolled patients, with a median of 4 (1–15) cycles per patient. There were 3 (13%) and 2 (9%) patients that had either dose delays or reductions, respectively. The median of relative dose intensity was 100% (range 64–100%). The median number of previous systemic therapy lines in the 23 enrolled patients was 3 (1–5), and 8 (34.8%) had received 4 or more lines. Other baseline characteristics are depicted in Table 1.

**Table 1:** Clinical and pathologic characteristics of accrued patients

	Accrued patients (*n* = 23)	Per-protocol population (*n* = 21)
Median age (Range)	49 (20–74)	57 (20–74)
*Gender*		
Male	13 (57%)	12 (57%)
Female	10 (43%)	9 (43%)
*Histology*		
Leiomyosarcoma	5 (22%)	5 (24%)
Others	18 (78%)	16 (76%)
*ECOG*		
0	10 (43%)	10 (48%)
1	13 (57%)	11 (52%)
*Extension at diagnosis*		
Localized	13 (57%)	11 (52%)
Locally advanced	6 (26%)	6 (29%)
Metastatic	4 (17%)	4 (19%)
*Extension at baseline*		
Locally advanced	2 (9%)	2 (9%)
Metastatic	21 (91%)	19 (90%)
*Location*		
Somatic	15 (65%)	13 (62%)
Visceral	8 (35%)	8 (38%)
Median primary size (range; mm)	95 (12–230)	95 (12–230)
Median previous surgery (range)	1 (0–13)	1 (0–13)
Median previous chemotherapy (range)	2 (1–5)	2 (1–5)
Median previous radiotherapy (range)	0 (0–4)	0 (0–4)
Median previous systemic (range)	3 (1–5)	3 (1–5)
Dose reduction	2 (9%)	2 (9%)
Treatment delay	5 (22%)	4 (19%)
*Best response*		
SD	13 (57%)	13 (62%)
PD	8 (35%)	8 (38%)
Non-evaluable	2 (9%)	–

**Fig. 2:**
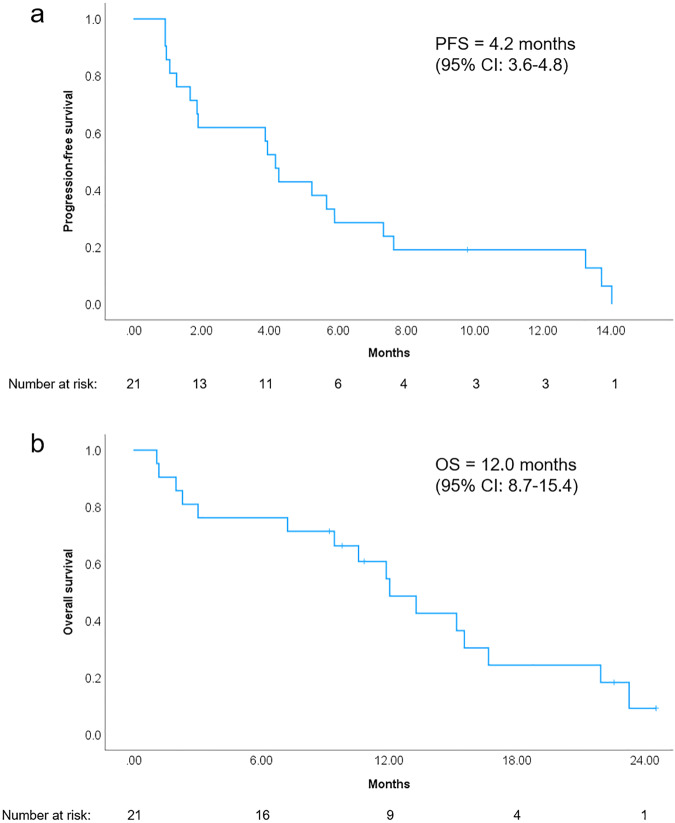
Survival analysis of per-protocol population (*n* = 21). **a** Progression-free survival and **b** overall survival

**Fig. 3:**
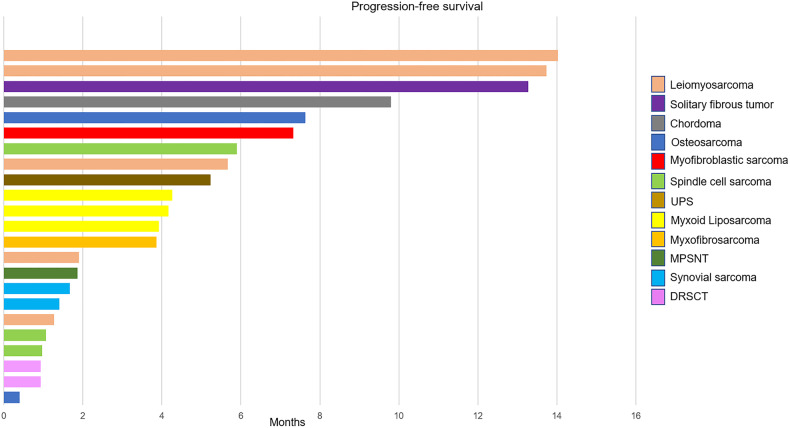
Swimmers plot illustrating progression-free survival of all individual patients in the study

Based on central radiology assessment at the time of clinical cutoff, the distribution of best RECIST responses was, stable disease (SD) 13 (62%), and progressive disease (PD) 8 (38%) for the per-protocol population. At a median follow-up period of 24 months, there were 20 events of progression and 16 events of death from the intent-to-treat population. The 6-m PFSR was 29% (95% CI 9–48), and there were 6 patients out of 21 with PFS longer than 6 months. The 12-m PFSR was 19% (95% CI 2–36). The median of PFS and OS was 4.2 (95% CI 3.6–4.8) and 12 (95% CI 8.7–15.4) months, respectively (Fig. 2). The longest PFS was seen in leiomyosarcoma, solitary fibrous tumor, and myofibroblastic sarcoma (Fig. 3).

Univariate analysis of clinical variables showed that female gender, ECOG PS 0, some shrinkage in target lesions, an interval between localized and advanced disease longer than 1 year and achieving stable disease were significant favorable prognostic factors for overall survival (Table 2).

**Table 2:** Univariate analysis of clinical and pathological factors of per-protocol population (*n* = 21)

	Median PFS (95% CI)	*p*	Median OS (95% CI)	*p*
*Age*		0.12		0.54
20–57	3.9 (1–6.7)		11.9 (6.7–17.1)	
>57	5.7 (3.1–8.2)		13.3 (6.1–20.3)	
*Sex*		0.13		0.045
Male	1.9 (0–5.6)		9.4 (4.3–14.6)	
Female	5.7 (4.4–6.9)		15.5 (14.6–16.4)	
*Histology*		0.18		0.072
Leiomyosarcoma	5.7 (0–13.7)		15.5 (14.7–16.3)	
Others	3.9 (3.3–4.5)		10.6 (7- 14.1)	
*ECOG*		0.89		0.014
0	4.2 (2.1–6.2)		21.9 (10.8–33.1)	
1	1.9 (0–5.1)		10.6 (0–22)	
*Extension at diagnosis*		0.44		0.96
Localized	4.3 (0.7–7.9)		11.9 (10–13.7)	
Locally advanced	3.9 (0.4–7.3)		16.7 (0–45.4)	
Metastatic	1 (0–5.6)		2.3 (0–14.1)	
*Extension at baseline*		0.79		0.96
Locally advanced	4.3 (NA)		10.6 (NA)	
Metastatic	3.9 (0.7–7.2)		12 (9.6–14.5)	
*Location*		0.51		0.65
Somatic	4.3 (3.7–4.9)		12 (5.5–18.5)	
Visceral	1.9 (0.7–3.2)		11.9 (0–27.1)	
*Primary size*		0.58		0.43
0–99	5.2 (1.2–9.3)		15.5 (13.4–17.6)	
>99	3.9 (0.5–7.4)		10.6 (4.9–16.2)	
*Best response*		<0.001		<0.001
SD	5.9 (3.4–8.4)		16.7 (11.7–21.6)	
PD	1.1 (0.6–1.5)		2.3 (0.9–3.7)	

There were no deaths related to adverse events. The most frequent secondary drug-related adverse events (AE) from treatment initiation (TEAEs) of any grade observed in the 23 patients, safety population, were neutropenia (60.9%), lymphopenia (47.8%), leukopenia (30.4%), anemia (21.7%) and fatigue (30.4%). The only grade 4 AE was a case of grade 4 lymphocytopenia. Apart from hematological events, grade 3 toxicities consisted of hypocalcemia (4.3%), creatinine increase (4.3%), and hypokalemia (4.5%) [Supplementary Table 1].

Of note, patients with mRNA tumor expression of *CDK4* higher than the median value had a tendency to a longer median PFS [(5.23 months, 95% CI 3.04–7.43) vs. (1.90 months, 95% CI 0.64–3.16), *p* = 0.11] and OS [(15.53 months, 95% CI 8.99–22.09) vs. (10.57 months, 95% CI 0.0–23.19), *p* = 0.098] (Table 3). Likewise, univariate analysis of selected biomarkers showed that the expression of CDK4 protein was a prognostic factor for OS of patients treated with palbociclib. Patients with high CDK4 expression (score 4–6) had better OS, compared to patients with low expression of CKD4 (score 2–3): 13.27 months (95% CI: 9.20–17.34) vs. 7.23 months (95% CI: NA), *p* = 0.034 (Table 3). The protein score of CDK4 per patient is shown in Supplementary Table 2. Notably, a significant correlation between *CDK4* mRNA levels and the immunohistochemical score of CDK4 protein expression levels was determined in our patients (*p* = 0.019) (Supplementary Table 3).

**Table 3:** Univariate analysis of potential prognostic/ predictive biomarkers

	*N*	Median PFS (95% CI)	*p*	Median OS (95% CI)	*p*
*CDK4* mRNA expression			0.11		0.098
Below median	11	1.90 (0.64–3.16)		10.57 (0–23.19)	
Above median	11	5.23 (3.04–7.43)		15.53 (8.99–22.09)	
*CDKN2A* mRNA expression			0.51		0.63
Below median	11	4.27 (0–8.59)		12.03 (0–24.55)	
Above median	11	3.93 (1.49–6.38)		13.27 (7.62–18.91)	
*RB1* mutation			0.22		0.95
Mutated	5	1.87 (0.58–3.15)		12.03 (11.67–12.39)	
Wild type	11	4.27 (2.79–5.74)		15.17 (6.47–23.86)	
*RB1* mutation (ACMG recommendations			0.067		0.26
Pathogenic	2	0.97 (NA)		2.3 (NA)	
Likely pathogenic	3	5.67 (0–12.71)		16.67 (9.25–24.08)	
Wild type	11	4.27 (2.79–5.74)		15.17 (6.47–23.86)	
*RB1* pathological			0.023		0.13
Yes	2	0.97 (NA)		2.3 (NA)	
No	14	4.27 (2.31–6.22)		15.17 (9.3–21.04)	
*TP53*			0.052		0.22
Mutated	4	1.07 (0.15–1.98)		2.3 (0–10.9)	
Wild type	12	4.27 (1.72–6.81)		12.03 (5.81–18.26)	
*RB1 and TP53*			0.020		0.15
Wild type	11	5.67 (3.8–7.54)		15.53 (8.95–22.11)	
*RB1* and/ or *TP53* mutated	5	1.87 (0.15–3.58)		11.87 (0–26.79)	
*CDK4* amplification			0.31		0.081
Yes	4	1.87 (1.05–2.68)		9.43 (0–19.1)	
No	12	4.27 (2.46–6.08)		15.53 (8.4–22.67)	
*CDKN2A* Loss			0.19		0.40
Yes	6	5.23 (2.79–7.67)		16.67 (14.23–19.1)	
No	11	3.93 (0.8–7.06)		10.57 (5.76–15.37)	
*CDK4 protein expression*			0.77		0.68
25–49%	8	3.87 (1.05–6.68)		15.17 (5.46–24.88)	
50–100%	13	5.23 (0–10.75)		11.87 (9.69–10.04)	
*CDK4 strength of immunostaining*			0.27		0.055
Weak	2	0.93 (NA)		1.2 (NA)	
Strong	19	4.17 (3.6–4.73)		12.03 (9.39–14.67)	
*CDK4 SCORE*^a^			0.59		0.072
2	2	0.93 (NA)		1.2 (NA)	
3	1	NR		NR	
4	6	3.87 (1.43–6.31)		15.53 (14.77–16.3)	
6	12	4.27 (0–9.98)		11.87 (9.68–14.06)	
*CDK4 SCORE*^a^			0.38		0.034
2–3	3	4.17 (NA)		7.23 (NA)	
4–6	18	3.93 (3.1–4.76)		13.27 (9.20–17.34)	
Cyclin E3 protein expression			0.59		0.87
Negative	1	NR		NR	
5–24%	2	1.9 (NA)		15.17 (NA)	
25–49%	6	3.87 (0–8.04)		11.87 (6.79–16.94)	
50–100%	12	3.93 (0–8.18)		10.57 (3.4–17.73)	
*Cyclin E3 strength of immunostaining*			0.79		0.48
Negative	1	5.23 (NA)		NR	
Weak	6	1.9 (1.83–1.97)		11.87 (6.79–16.94)	
Strong	14	3.93 (3.38–4.48)		12.03 (7.5–16.56)	
*Cyclin E3 SCORE*^a^			0.67		0.46
Negative	1	NR		NR	
2	2	1.9 (NA)		15.17 (NA)	
3	4	1.87 (0.47–3.26)		9.43 (0–21.76)	
4	2	3.87 (NA)		13.27 (NA)	
6	12	3.93 (0–8.18)		10.57 (3.4–17.73)	
*E2F1 protein expression*			0.98		0.31
5–24%	7	5.23 (4.04–6.42)		13.27 (7.51–19.02)	
25–49%	7	1.87 (0.33–3.41)		11.87 (5.62–18.11)	
50–100%	7	3.93 (0–9.15)		21.93 (1.4–42.46)	
*E2F1 strength of immunostaining*			0.92		0.043
Negative	7	5.23 (4.04–6.42)		13.27 (7.51–19.02)	
Weak	8	1.87 (1.54–2.19)		9.43 (0–19.77)	
Strong	6	3.87 (0–9.15)		21.93 (11.15–32.72)	

^a^Considering both the expression and the strength of immunostaining

The series was *a posteriori* screened for *RB1* and *TP53* gene mutations, among other biomarkers (Supplementary Table 4). *RB1* and *TP53* were found to be mutated, with a pathogenic mutation according to ACMG recommendations, in 2 and 4 patients, respectively. Notably, the two *RB1* mutated cases presented a significantly worse PFS [0.97 months (95% CI NA) vs. 4.27 months (95% CI 2.31–6.22) (*p* = 0.023)], whereas *TP53* wild type showed a good trend for better PFS [4.27 months (95% CI: 1.72–6.81) vs. 1.07 months (95% CI: 0.15–1.98), *p* = 0.052] (Table 3). Patients with wild type *TP53* and *RB1* had significantly better PFS, compared with patients with any mutation in any of these genes: 5.67 months (95% CI: 3.80–7.54) vs. 1.87 months (95% CI 0.15–3.58), *p* = 0.020 (Supplementary Fig. 1).

## Discussion

This phase II trial met its primary endpoint of 6-month PFSR with 6 out of 21 evaluable patients (29%) achieving a PFS longer than 6 months. This outcome was obtained in a wide range of histological sarcoma subtypes, with the only exclusions being well-differentiated/dedifferentiated liposarcoma (WD/DD-LPS), rhabdomyosarcoma and Ewing sarcoma. The median PFS of 4.2 months and OS of 12 months reached for patients with progressing tumors at baseline and having been heavily pretreated, with a median of 3 previous systemic therapeutic lines, is encouraging. This outcome seems favorable to a real-life setting, where in a similar pretreated subset of sarcoma patients the time to next treatment, which can be equated with PFS, ranged from 2.3 to 3.7 months and the OS ranged from 5.4 to 8.5 months.^21^ Moreover, the survival outcomes obtained in our study also compare with those observed in the pivotal studies of active drugs in second-line sarcomas. Pazopanib, trabectedin, and eribulin studies reported a median of PFS and OS that ranged from 2.6–4.6 months and 12.4–13.5 months, respectively.^22–24^

The most recognized consequence of CDK4/6 inhibition is RB-dependent proliferative arrest. Palbociclib prevents the phosphorylation of the Rb protein and, as a result, prevents its inactivation. As expected, this cytostatic effect would attain tumor stabilization at best with uncommon tumor responses. The proportion of stabilizations detected (62%) also favorably compares with the clinical benefit rate reported in third (less than 50%) or further lines (<25%) in advanced STS.^25^

Patient selection by high mRNA expression of *CDK4*, while *CDKN2A* was not overexpressed at baseline tumor followed an extensive investigation. The first condition was to replicate the in vivo preclinical experience. Thus, Stratagene RNA human pool was the best reference for replicating the biomarker conditions for success and failure in the palbociclib treatment of PDX sarcoma models and to correlate with protein expression. The second condition was simplicity: any overexpression of *CDK4* and equal or underexpression of *CDKN2A* for the tested sarcoma against the RNA pool, accomplished with the biomarker selection. Lastly, the selection of a commercial RNA pool makes the relative RNA expression reproducible. *A posteriori* analysis of protein expression for CDK4 supported the RNA *CDK4* selection. On the one hand, the high protein expression for CDK4 (scores 4–6), detected in 86% of cases, corroborated an adequate selection based on overexpression of *CDK4*. On the other hand, there was a significant correlation between CDK4 protein score and *CDK4* RNA expression, reinforcing the fact that our series overexpressed CDK4. A further question is whether the threshold for *CDK4* overexpression should be fine-tuned. The fact that patients with tumor expression of *CDK4* above the median value showed a longer PFS and OS would suggest there is a margin for improvement in the *CDK4* cutoff. The results of our study also suggest that the CDK4 IHC protein score may be an adequate biomarker for CDK4 inhibitor activity in sarcoma, in addition to CDK4 mRNA expression. This observation should be further tested in future translational and clinical research.

Besides, the unplanned posterior mutation screening for *RB1* and *TP53* genes interestingly revealed that the two patients harboring a pathological mutation of *RB1* evidenced a significantly shorter PFS, whereas the four patients with tumors carrying pathological *TP53* mutations showed worse PFS. The academic nature of the study did not allow us to assume the logistics for mutation screening of these genes for patient accrual. However, in tumors without proficient RB CDK4/6 inhibition is not effective.^26^ Furthermore, *TP53* mutations appeared to be strongly related to CDK4/6 inhibitor resistance in a panel of 560 cancer cell lines treated with palbociclib and abemaciclib.^27^ This is in line with our findings and could explain the worse PFS observed in the *TP53* mutated cases. The lack of paired tumor samples after the initiation of CDK4/6 inhibition precludes us from excluding the tumor transformation towards a senescence-like phenotype, where proficient RB would be more relevant than p53.^28^

Amplification of CDK4 is one molecular hallmark of WD/DD-LPS and is the reason why palbociclib was tested in this specific sarcoma subtype. In two different studies that enrolled 29 and 60 evaluable advanced WD/DD-LPS patients, palbociclib was prescribed at 200 mg per day, 2 weeks on and one week off, and 125 mg per day, 3 weeks on and 1 week off, respectively.^16,29^ Both trials reported an identical median PFS of 4.2 months that was superimposable to our study, reinforcing the idea that *CDK4* overexpression was a reasonable strategy of biomarker selection for CDK4/6 inhibition in sarcoma. Ribociclib was tested in a phase I trial accruing 30 DD-LPS patients, among other solid tumors, with six LPS patients demonstrating SD at 6 months on treatment, comparable to our study. Abemaciclib showed higher disease control with a median PFS of 7 months in a phase II trial enrolling advanced DD-LPS patients.^30^ We found four individuals with CDK4 tumor amplification in our study but there was either no correlation with CDK4 expression or with a better survival outcome. Even when it is generally accepted as synonymous, gene amplification is not always equivalent to overexpression.^31^ Furthermore, in some tumor contexts CDK4 amplification was related to resistance to CDK4 inhibition.^32^

In recent times, several works have emphasized the relevance of the p16-CDK4/6-RB1 pathway in sarcomas. Alteration of the *CDKN2A* gene was the only finding associated with worse OS across all sarcoma subtypes in a series of 7,733 sarcoma cases analyzed with next-generation sequencing (Foundation Medicine). *CDKN2A* alterations were detected in 22% of sarcoma cases.^33^ When broken down into different histologic subtypes other than DD-LPS, several findings are worthy of mention among the most frequent sarcomas. In UPS, gains in chr12q13-15 that contains the *CDK4* gene have been found in up to 30% of cases.^34^ Activation of Ras signaling, commonly seen in UPS, results in increased transcription of CDK4/6 also linked to UPS development.^35^ *CDK4* amplification was detected in up to 27.8% in some small series of LMS and authors corroborated the efficacy of palbociclib in LMS cell lines with similar biomarker conditions to our study.^36^ *CDKN2A* deletions were detected in 21% of LMS and correlated with poor OS.^37^ Interestingly, three out of three patients with LMS of soft tissue in our study had a PFS longer than the median value, and two of them had a PFS more than three times the median PFS of the series (14 and 13.7 months). In synovial sarcoma, CDK4 is highly expressed and high CDK4 expression was associated with a poor prognosis.^38^ A genetic disruption of *CDKN2A* is frequently detected in malignant peripheral nervous sheath tumors (MPNST) that elicits the de-repression of *CCND1* and *CCNE1* and could drive the MPNST progression.^39^ Genomic data highlight that the *CDKN2A*-*CDK4/6*-*RB1* pathway is affected in more than 25% of sarcomas and represents a key oncogenic driver.^13,40^

The lack of *RB1* sequencing at baseline and the challenge of obtaining tumor biopsy for genomic screening purposes in the context of advanced sarcoma patients, additionally hindered by the SARS-CoV2 pandemic, have been limitations of the trial. Furthermore, it cannot be excluded the impact of the limited number of accrued patients and the indolent behavior inherent to some subtypes on the clinical outcome. Indeed, it would be valuable to determine the clinical outcome, in terms of PFS and OS, of an advanced and progressing independent sarcoma population exhibiting overexpression of *CDK4*, without overexpression of *CDKN2A*.

The study offered RNA analysis for *CDK4* and *CDKN2A* in the archival tumor material with the assumption that if a biomarker expression fitting with the inclusion criteria was found, then the screening at baseline would render a highly probable similar RNA expression. As a matter of fact, 41% of those with a suitable biomarker for the study inclusion criteria in the archival material were also suitable at the biopsy performed at baseline. This proportion is not substantially higher than direct screening where 29% of patients exhibited a suitable biomarker expression profile for the study.

Despite the fact that a valuable predictive biomarker selection for CDK4/6 inhibition is challenging, the selected biomarkers with RNA overexpression of *CDK4* and no overexpression of *CDKN2A* seem adequate for future studies with CDK4/6 inhibitors in sarcomas. Evidently, sequencing for discarding pathogenic mutations in the *RB1* gene is critical, and probably doing so for the *TP53* gene is also important according to our results. Considering the variances observed in the expression of CDK4 in pre-screening and screening tissue, this quadruple biomarker determination is recommended in screening biopsies. Of note, considering that 16 patients fitting the 4 biomarker conditions (overexpression of *CDK4*, no overexpression of *CDKN2A*, and no pathogenic mutations in RB1 or *TP53*) exhibited improved outcome with a median PFS of 5.67 months and a median OS of 15 months, this 4-biomarker set should be validated in prospective studies testing CDK4/6 inhibitors in sarcomas other than WD/DD-LPS. Protein expression of CDK4 scoring 4 or higher would seem to be equivalent to the RNA overexpression cutoff we selected with the Stratagene RNA pool and should also be prospectively validated.

Future therapeutic strategies combining CDK4/6 inhibitors with other compounds seem worthwhile and also deserve to be explored in the sarcoma field. The synergy between CDK4/6 inhibitors and estrogen receptor (ER) antagonists in blocking ER^+^ breast tumor proliferation,^41^ led to positive clinical trials and constitutes a good model for improving clinical outcomes by combining CDK4/6 inhibitors.^42^ Targeting the bromodomain and extraterminal (BET) family of proteins synergized with CDK4/6 inhibitors in osteosarcoma cell lines in an MYC-independent way.^43^ The combination of regorafenib and palbociclib was shown to be highly effective in osteosarcoma PDX models.^44^ Direct mTOR overexpression or indirect PTEN loss is more frequently seen in some sarcomas, such as angiosarcoma, leiomyosarcoma, and osteosarcoma. The combination of mTOR and CDK4/6 inhibitors is being tested in a phase II trial in DD-LPS and LMS, supported by preclinical rationale.^45^

The findings from this single-arm, phase II trial suggest that palbociclib has activity in advanced sarcomas other than WD/DD-LPS selected by RNA expression of *CDK4* and *CDKN2A*, showing a promising median PFS and OS in a heavily pretreated population. A significant correlation between median PFS, OS, and the overexpression level of *CDK4* indicates that CDK4/6 inhibitors may have a role in the treatment of advanced sarcoma patients. A fine-tuned selection with a 4-biomarker set is recommended based on this trial, for future use of CDK4/6 inhibitors in sarcoma patients.

## Material and methods

### Study design and participants

In this single-arm phase II trial, adult patients (aged ≥18 years) bearing advanced and measurable tumors according to RECIST criteria with a diagnosis of any sarcoma subtype (excluding DD-LPS, rhabdomyosarcoma, and Ewing sarcoma) that had progressed in the last 6 months, were screened to participate at 13 tertiary Spanish hospitals with expertise in sarcoma care and research. Additional inclusion criteria were central pathology confirmation of sarcoma, overexpression of *CDK4* and no overexpression of *CDKN2A* in the tumor carried out by a central laboratory (more details below, in the specific block of the methods section), ECOG performance status of 0–1 and adequate bone marrow, liver, cardiac, renal and pulmonary function. Some additional relevant exclusion criteria were previous treatment with cell-cycle checkpoint inhibitors, more than 3 previous systemic lines for advanced disease, metastasis in the central nervous system, pregnancy, breastfeeding, concomitant prescription of CYP3A4 substrates with a narrow therapeutic window or causing QT interval prolongation, and radiotherapy in target lesions, among others. The main endpoint was PFS rate at 6 months (6-m PFSR), while secondary endpoints were ORR according to RECIST 1.1 and Choi criteria, median of PFS, median of OS, safety profile according to CTCAE 4.0 and correlation between translational variables and clinical outcome. Procedures were conducted in accordance with guidelines established by the local ethics committee from each hospital, after approval from these institutions, and in accordance with the Declaration of Helsinki. Informed consent was obtained from each participant before the activation of the screening process.

### CDK4 and CDKN2A cut-off reference for RNA overexpression

For determining tumoral RNA overexpression of *CDK4*, while no overexpression of *CDKN2A*, we carried out qRT-PCR for these two genes, using RNA extracted from different RNA pools and compared the outcome by its reproducibility and robustness among independent determinations and tumor samples; a commercial pool of cancer cell lines (#750500, Stratagene QPCR Human Reference Total RNA; (Agilent®), Santa Clara, CA, USA) was selected as the best reference as cut-off point (Supplementary Fig. 2). Thereby, any tumoral RNA expression exceeding the average of the pool values for *CDK4* and *CDKN2A* was considered overexpression. The reference RNA pool was included in parallel as control for each sample tested and the average of triplicate analysis in each determination was defined as the cut-off. A baseline biopsy for determining *CDK4/ CDKN2A* expression was mandatory unless tumor samples were available within 3 months before enrolment, and no treatment was administered in this time period.

Additionally, the performance of a pre-screening of RNA expression for both genes in archival tumor samples was allowed, under the presumption that if in line with the inclusion criteria, there would be a higher probability of complying with the RNA expression values for the accrual in the baseline biopsy.

### Patient screening

Total RNA was isolated from FFPE tumor blocks (starting from 10 cuts of 10 µm), using the Recover All Total Nucleic Acid Isolation® kit (Ref: AM1975; Ambion, Thermo Fisher Scientific, Waltham, MA, USA), following manufacturers’ instructions. The cDNA synthesis was performed using 200 ng of RNA, random primers, a dNTP mix, and MultiScribe Reverse Transcriptase in a total volume of 25 μL (Ref: 4368814; High Capacity cDNA Reverse Transcription Kit; Applied Biosystems, Waltham, MA, USA). The qPCR reactions were performed in 384-well plates with the TaqMan Gene Expression Assays (Applied Biosystems). To quantify gene expression the following TaqMan^TM^ probes from Applied Biosystems were used: human *CDK4* (Hs_01565683_g1), human *CDKN2A* (Hs_00923894_m1) and human *GAPDH* as endogenous control (Hs03929097_g1). The relative mRNA quantities were expressed as log10RQ (Relative quantification). Relative mRNA quantification and statistical analysis of qPCR data were conducted using RQ Manager 1.2.1 software (Applied Biosystems). All of the screening batches included the *Stratagene QPCR Human Reference Total RNA* as external standard control to relativization and positive control with overexpression of *CDK4* and low expression of *CDKN2A*. All screenings were performed with three experimental replicates.

The mRNA expression levels of both *CDK4* and *CDKN2A* were also used for translational purposes to determine the prognostic/predictive value of both genes.

### Immunohistochemistry

The protein expression levels of CDK4, cyclin E, and E2F1 were assessed by immunohistochemistry (IHC) in formalin-fixed paraffin-embedded (FFPE) samples to determine their prognostic/predictive value. Four-μm sections were cut from the FFPE and stained with hematoxylin and eosin and with immunohistochemistry staining. Immunohistochemistry used an anti-CDK4 mouse monoclonal antibody (sc-56277; Santa Cruz Biotechnology, Inc., Dallas, TX, USA), an anti-cyclin E mouse monoclonal antibody (sc-247; Santa Cruz Biotechnology, Inc.) or an anti-E2F1 rabbit polyclonal antibody (#3742; Cell Signaling Technology, Danvers, MA, USA). The extension of protein expression was evaluated in 4 levels (0—negative; 1—extension between 5% and 24%; 2—extension between 25% and 49%; and 3—extension between 50% and 100%) and the strength of IHC was evaluated in 3 levels (0—negative; 1—weak; and 2—strong). An immunohistochemical score was determined for CDK4 (CDK4 score) expression levels, by multiplying the extension of protein expression (level 0–3) by the strength of IHC (level 0–2). The CDK4 score was grouped as low expression (score of 2–3) and high expression (score of 4–6) for statistical analysis. A pathologist with great expertize in sarcomas (RR) was responsible for reviewing protein expression, blinded to clinical data.

### Copy number variation (CNV) and single nucleotide variant (SNV) analysis

DNA was extracted from FFPE tumor blocks, using the QIAamp^®^ DNA FFPE Tissue Kit (QIAGEN, Hilden, Germany), following the manufacturer’s protocol. The DNA concentration of each sample was analyzed by fluorimetric quantification with the Qubit system (Thermo Fisher Scientific Inc., Waltham, MA, USA), while the purity degree was evaluated by spectrophotometry, using a NanoDrop system (Thermo Fisher Scientific Inc.), and the integrity of the DNA by means of TapeStation (Agilent Technologies). Sequencing libraries were generated from the fragmented genomic DNA (150–200 bp) with the SureSelectXT Custom Constitutional Panel 17 Mb (ref. 5191-4059; Agilent), using Covaris (Covaris, Woburn, MA, USA). The quality of the amplified libraries was evaluated using TapeStation (Agilent Technologies). The genomic regions of interest were captured using RNA probes (SureSelectXT) from the amplified libraries. Captured libraries were consequently indexed, amplified, and purified and their quality was once again determined using TapeStation (Agilent Technologies). The generated libraries were normalized and pooled at equimolar concentrations for optimal generation of DNA clusters. SureSelectXT Custom Constitutional Panel 17 Mb libraries were sequenced on the NovaSeq 6000 platform (Illumina, Inc., San Diego, CA, USA). Variant calling was identified with Illumina DRAGEN Bio-IT Platform (Illumina). Single nucleotide variants (SNVs) annotations were determined using software developed by NIMGENETICS (Madrid, Spain) that mixes public and proprietary databases, and copy number variations (CNVs) annotations were determined with AnnotSV.^46,47^ The pathogenicity of sequence variants was determined following the recommendations of the American College of Medical Genetics and Genomics (ACMG).^48^ The pathogenicity of *TP53* variants was determined with COSMIC (cancer.sanger.ac.uk).^49^

### Clinical procedures

Patients received palbociclib 125 mg once daily for 21 consecutive days, followed by 7 days off in 28-day cycles. Palbociclib should be swallowed whole and taken with food. Dose reductions or interruptions were established according to the drug brochure. Hematological grade 3 or 4 toxicity on day 1 required a delay of one further week, or longer, up to recovery ≤grade 2. In the event of grade 3 neutropenia lasting more than one week or recurrent grade 3 on day 1, dose reduction was considered. Two dose-level reductions, up to 100 and 75 mg per day, were allowed, whereas a further dose reduction would lead to treatment cessation. In the case of non-hematological grade 3 toxicities, treatment had to be postponed up to recovery ≤grade 2, and palbociclib was then resumed with an inferior dose level. Treatment with palbociclib was continued until any of the following events occurred: disease progression, unacceptable toxicity, withdrawal of consent, a requirement for a third dose reduction, or if the patient was considered by the sponsor or investigator to be non-compliant with the requirements of the protocol. Central pathology confirmation was required before the accrual. Central radiology review was also compulsory, but it was planned to be performed at the time of the end of the study. Appropriate imaging tests usually by CT scan and, occasionally, by MRI were performed every 8 weeks and anonymously uploaded to a web-based imaging platform.

### Statistical analysis

Sample size was obtained with Simon’s two-stage design for the primary endpoint of a 6-month progression-free survival rate, and estimated accrual of 24 months. The initial design estimated a 6-m PFSR of 14%, the EORTC cut-off for second-line active drugs,^50^ as not promising, whereas one of 35% would be considered promising in this population. With a type I error of 0.1 and a power of 0.90, patient numbers were estimated at 38 for this trial. In the first stage, at least 3 cases of the first 15 patients should have ≥6-m PFS. If this occurred, an additional 23 patients would be accrued in the second stage of the trial up to a total of 38 patients. To reject the null hypothesis, at least 9 patients should have a 6-m PFS or longer.

An amendment was approved increasing the delta value between H0 and H1 due to a slower accrual related, in particular due to the COVID pandemic. In the new design, the type I and II errors (10%) remained unchanged, but the null hypothesis (H0) was increased up to 15%, while the alternative hypothesis (H1) was increased up to 40%. With Simon’s two-stage Minimax design, 21 patients were estimated. At least 3 cases of the 15 first patients (first stage) should have ≥6-m PFS. Then, an additional six evaluable patients would be accrued (second stage) and to reject the null hypothesis at least six patients should have a 6-m PFS or longer.

Those patients who provided written consent, whose tumors were centrally confirmed for the histotype and molecular expression, formed the intention-to-treat population. The per-protocol population was defined as the subset of the intention-to-treat population with measurable disease at study entry (as per RECIST criteria). Patients in this population also received at least one month (one cycle) of treatment and had at least one radiological assessment. Otherwise, the patient was not considered assessable (the exception was early progression or death, for which patients were included). Patients who received at least one dose of palbociclib were considered evaluable for toxicity.

Variables following binomial distributions (i.e. response rate), were expressed as frequencies and percentages. Comparisons between qualitative variables were done using the Fisher Exact Test or Chi-square. Comparisons between quantitative and qualitative variables were performed through non-parametric tests (*U* of Mann–Whitney or Kruskal–Wallis). Time-to-event variables (OS and PFS) were measured from the date of trial enrollment and were estimated according to the Kaplan–Meier method. Comparisons between the variables of interest were performed using the log-rank test. All *p*-values reported were two-sided, and statistical significance was defined at *p* < 0.05.

### Supplementary information

Supplementary material

Study protocol
