# Supplementary material for: Phase II trial of CDK4/6 inhibitor palbociclib in advanced sarcoma based on mRNA expression of CDK4/CDKN2A
Source: Signal Transduct Target Ther. 2023 Oct 25;8:405. doi: 10.1038/s41392-023-01661-8 (PMC10598203; doi:10.1038/s41392-023-01661-8)
Supplement: Supplementary file 1 — Supplementary material [file 41392_2023_1661_MOESM1_ESM.docx]

Supplementary Materials for

Phase II trial of CDK4/6 inhibitor palbociclib in advanced sarcoma based on mRNA expression of *CDK4/ CDKN2A*

Javier Martin-Broto^1,2,3^, Jeronimo Martinez-Garcia^4^, David S. Moura^1^, Andres Redondo^5^, Antonio Gutierrez^6^, Antonio Lopez-Pousa^7^, Javier Martinez-Trufero^8^, Isabel Sevilla^9^, Roberto Diaz-Beveridge^10^, Maria Pilar Solis-Hernandez^11^, Amancio Carnero^12^, Marco Perez^12,13^, David Marcilla^13^, Jesus Garcia-Foncillas^1,2^, Pablo Romero^1^, Javier Fernandez-Jara^14^, Daniel Lopez-Lopez^12,15,16^, Ivan Arribas^17^, Nadia Hindi^1,2,3^

*Corresponding author:* [*jmartin@atbsarc.org*](mailto:jmartin@atbsarc.org)

This file contains:

- Supplementary Figure 1 and 2
- Supplementary Table 1 to 4

Supplementary Figure 1


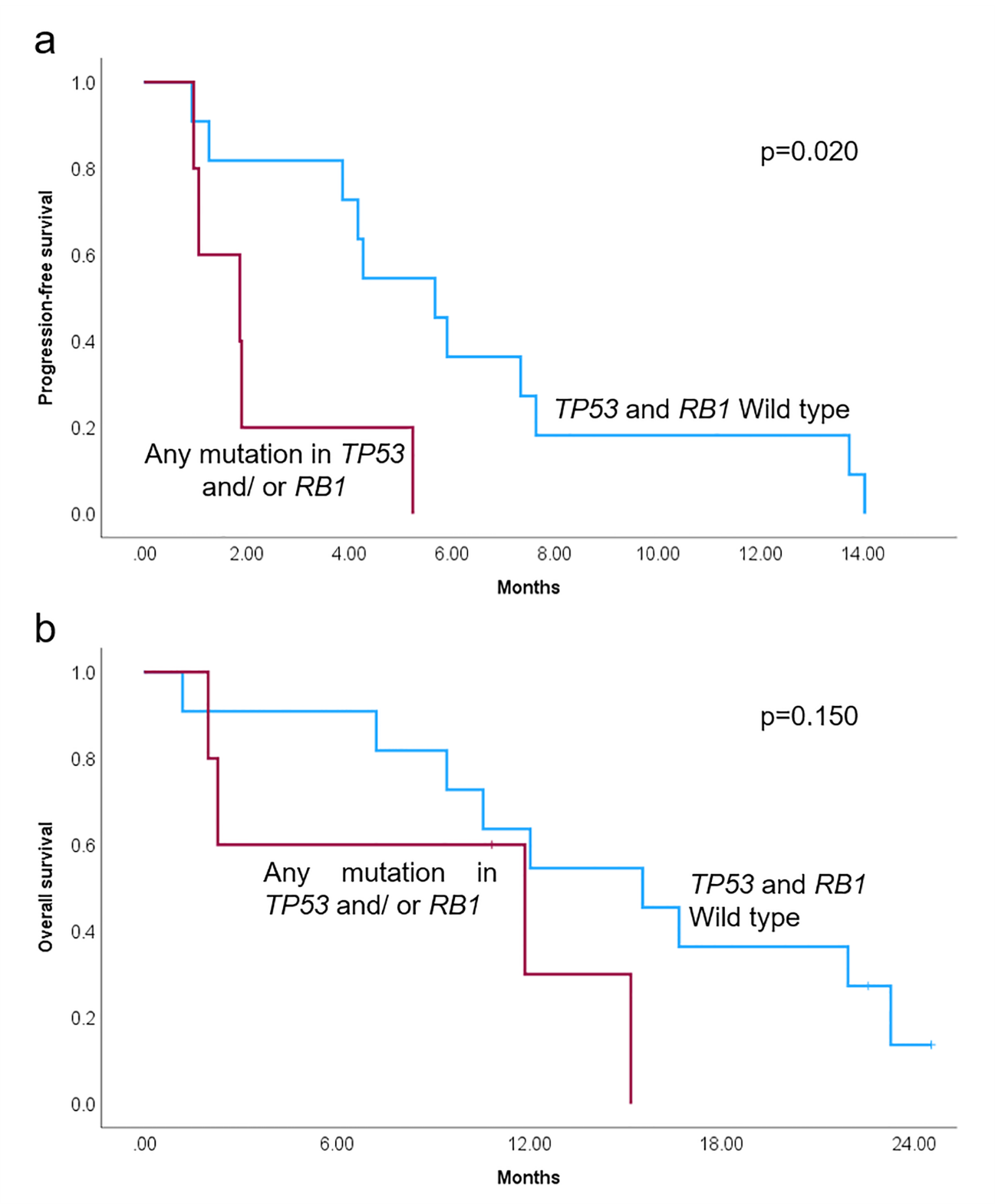


**Supplementary Figure 1. Survival of analysis according to TP53 and RB1 mutational status**. A) Progression-free survival and B) overall survival.

Supplementary Figure 2


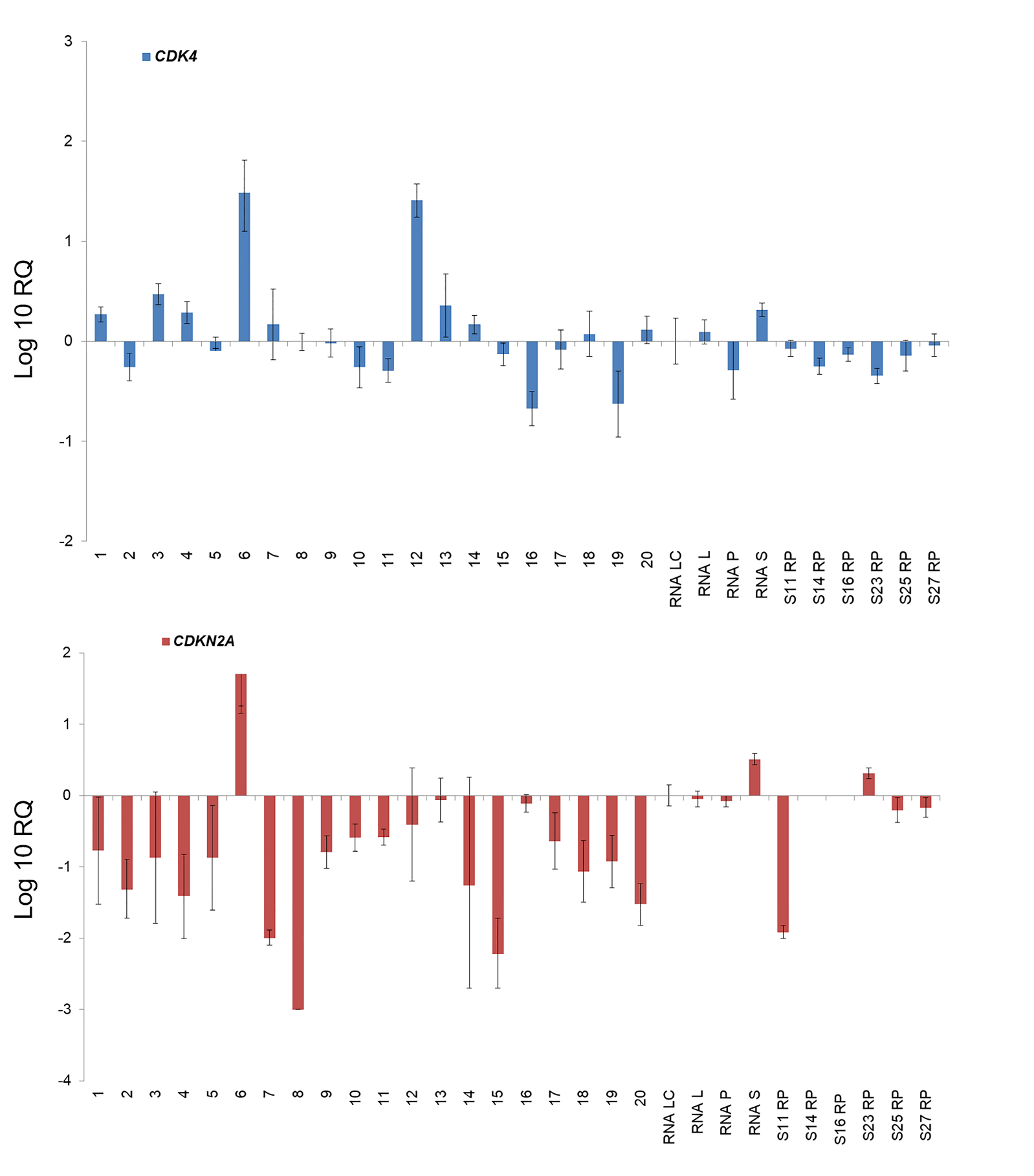


**Supplementary Figure 2. Assessment of the best external standard control to determine *CDK4* and *CDKN2A* (p16) expression levels by qRT-PCR and patient enrolment.** 1-20: GEIS-20 Tumor samples; RNA LC: Commercial Human Reference RNA; RNA L: RNA extracted from a pool of 13 sarcoma cell lines; RNA P: RNA extracted from a pool of FFPE of sarcomas; RNA S: RNA extracted from a pool of sarcomas fresh/frozen tissue; S11RP-S14RP-S16RP-S23RP-S25RP-S27RP: PDX models of sarcoma.

Supplementary Table 1. Safety profile (n=23)

| TERM | Any grade | Grade 1-2 | Grade 3 | Grade 4 |
| --- | --- | --- | --- | --- |
| Hematological |  |  |  |  |
| Neutropenia | 14 (60.9%) | 9 (39.1%) | 5 (21.7%) | 0 |
| Lymphocytopenia | 11 (47.8%) | 9 (39.1%) | 1 (4.3%) | 1 (4.3%) |
| Leukopenia | 7 (30.4%) | 6 (26.1%) | 1 (4.3%) | 0 |
| Anemia | 5 (21.7%) | 5 (21.7%) | 0 | 0 |
| Thrombocytopenia | 2 (8.7%) | 0 | 2 (8.7%) | 0 |
| Non-Hematological |  |  |  |  |
| Fatigue | 7 (30.4%) | 7 (30.4%) | 0 | 0 |
| Anorexia | 2 (8.7%) | 2 (8.7%) | 0 | 0 |
| Constipation | 2 (8.7%) | 2 (8.7%) | 0 | 0 |
| Nausea | 2 (8.7%) | 2 (8.7%) | 0 | 0 |
| Arthralgia | 1 (4.3%) | 1 (4.3%) | 0 | 0 |
| Dyspnea | 1 (4.3%) | 1 (4.3%) | 0 | 0 |
| Hypoalbuminemia | 1 (4.3%) | 1 (4.3%) | 0 | 0 |
| Hypocalcemia | 1 (4.3%) | 0 | 1 (4.3%) | 0 |
| Hypokalemia | 1 (4.3%) | 0 | 1 (4.3%) | 0 |
| Mucositis oral | 1 (4.3%) | 1 (4.3%) | 0 | 0 |
| Pancreatitis | 1 (4.3%) | 0 | 1 (4.3%) | 0 |
| Pyrosis | 1 (4.3%) | 1 (4.3%) | 0 | 0 |
| Vomiting | 1 (4.3%) | 1 (4.3%) | 0 | 0 |

Supplementary Table 2 – CDK4 protein expression score by patient

| Patient ID | Subtype | CDK4 IHC score |
| --- | --- | --- |
| 01 | Myxofibrosarcoma | 4 |
| 02 | Leiomyosarcoma | 6 |
| 03 | Leiomyosarcoma | 4 |
| 04 | Leiomyosarcoma | 4 |
| 05 | MPNST | 6 |
| 06 | Spindle cell sarcoma | 6 |
| 07 | DSRCT | 2 |
| 08 | Spindle cell sarcoma | 4 |
| 09 | Solitary fibrous tumor | 6 |
| 10 | Myofibroblastic sarcoma | 6 |
| 11 | Osteosarcoma | 6 |
| 12 | Synovial sarcoma | 6 |
| 13 | Synovial sarcoma | 3 |
| 14 | Myxoid liposarcoma | 2 |
| 15 | DSRCT | NA |
| 16 | Osteosarcoma | 6 |
| 17 | Leiomyosarcoma | 6 |
| 18 | Leiomyosarcoma | 4 |
| 19 | Myxoid liposarcoma | 6 |
| 20 | Spindle cell sarcoma | 6 |
| 21 | UPS | 6 |
| 22 | Chordoma | 6 |
| 23 | Myxoid liposarcoma | 4 |

DSRCT: Desmoplasic small round cell tumor; IHC: Immunohistochemistry; MPNST: Malignant peripheral nerve sheath tumor: NA: Not available; UPS: Undifferentiated pleomorphic sarcoma.

Supplementary Table 3. Correlation between *CDK4* mRNA expression and CDK4 protein expression.

| CDK4 Score* | Median mRNA  (Range) |
| --- | --- |
| 2-3 | 1.2735  (1.117-1.785) |
| 4-6 | 1.884  (1.1132-25.6) |
| U de Mann Whitney: p = 0.019 | |

*considering both the extension and strength of the immunostaining

Supplementary Table 4 – Copy number variation (CNV) and single nucleotide variant (SNV) profiling

| Patient ID | *CDK4* | *CDKN2A* | *RB1* | *TP53* |
| --- | --- | --- | --- | --- |
| 01 | Wild type | Deletion | Wild type | Wild type |
| 02 | Wild type | Wild type | Deletion^1^ | Wild type |
| 03 | Wild type | Deletion | Wild type | Wild type |
| 04 | Amplification | Wild type | Wild type | c.686G>A |
| 05 | Amplification | Deletion | Deletion^2^ | Wild type |
| 06 | Wild type | Deletion | Deletion^1^ | Wild type |
| 07 | Wild type | Wild type | Wild type | Wild type |
| 08 | Wild type | Wild type | Deletion^2^ | c.590T>G |
| 09 | NA | NA | NA | NA |
| 10 | Amplification | Wild type | Wild type | Wild type |
| 11 | Wild type | Deletion | Wild type | Wild type |
| 12 | NA | NA | NA | NA |
| 13 | NA | NA | NA | NA |
| 14 | Wild type | Wild type | Wild type | Wild type |
| 15 | NA | NA | NA | NA |
| 16 | NA | NA | NA | NA |
| 17 | Wild type | Wild type | Wild type | Wild type |
| 18 | Wild type | Wild type | Deletion^1^ | Wild type |
| 19 | Wild type | Wild type | Wild type | Wild type |
| 20 | Amplification | Wild type | Wild type | c.404G>T |
| 21 | Wild type | Deletion | Wild type | c.440T>C |
| 22 | Wild type | Deletion | Wild type | Wild type |
| 23 | NA | NA | NA | NA |

NA: Not available. ^1^Likely pathogenic and ^2^Pathogenic following the recommendations of the American College of Medical Genetics and Genomics (ACMG).
