# Supplementary material for: Phase II trial of CDK4/6 inhibitor palbociclib in advanced sarcoma based on mRNA expression of CDK4/CDKN2A
Source: Signal Transduct Target Ther. 2023 Oct 25;8:405. doi: 10.1038/s41392-023-01661-8 (PMC10598203; doi:10.1038/s41392-023-01661-8)
Supplement: Supplementary file 2 — Study protocol [file 41392_2023_1661_MOESM2_ESM.pdf]

GEIS-51

CLINICAL TRIAL PROTOCOL

Title:
Multicenter phase II trial of palbociclib in second line of advanced sarcomas with CDK4 overexpression

Sponsor's protocol code: GEIS-51
EudraCT number: 2016-004039-19
Acronym: PalboSarc

Protocol version: 4 of 29/04/2022

Sponsor: Grupo Español de Investigación en Sarcomas (GEIS)

Clinical coordinating investigators:

- Dr. Javier Martín – Hospital Universitario Fundación Jiménez Díaz (Madrid)
- Dr. Roberto Díaz – Hospital Universitari i Politècnic La Fe (Valencia)

Translational substudy coordinators:

- Dr. Amancio Carnero – Hospital Universitario Virgen del Rocío (Seville)
- Dr. Marco Pérez – Hospital Universitario Virgen del Rocío (Seville)
- Dr. David da Silva Moura – Instituto de Investigación Sanitaria Fundación Jiménez Díaz (Madrid)

All the information contained in this document is confidential and should not be disclosed to any person without the written consent of the Sponsor, unless the information is given to obtain the informed consent of the persons entering the study. The information may be disclosed for communications with the competent authorities, ethics committees or persons involved in the study.

VERSION HISTORY AND CHANGES TO THE PROTOCOL

Version	Date	Substantial modification no.	Summary of changes
1.0	15/10/2016	NA	• Initial protocol
2	23/06/2017	1	• Protocol version and date updated: p. 1, 5<br>• CRO contact information updated: pp. 3, 26, 31, 33 and 44<br>• Table of procedures and evaluations on pp. 13 and 24:<br>○ A margin of +/-7 days is indicated for the end-of-treatment and follow-up visits<br>○ The expression "Molecular review" is replaced by "CDK4 overexpression analysis"<br>○ A margin of +/-3 days for laboratory tests is indicated<br>○ Updated legends: (1) Pre-screening test, (4) Biochemistry, (5) Hematology, (6) Pregnancy test<br>• It is indicated that patients who fail the prescreening test are considered screen failures on p. 19 (section 4.4)<br>• New wording of sections 7.2 and 7.3<br>• Frequency of every 3 cycles for the pregnancy test is specified on p. 28 (section 7.5)<br>• New presentation (in table form) of the biological samples of the study on p. 42 (section 12.3)<br>• Change in the blood tubes used on p. 44 (section 12.4.2)
3	23/06/2018	2	• Protocol version and date updated: p. 1, 5<br>• Change in the inclusion criterion 8 and exclusion criterion 5 on pp. 10-11 (section 1) and p. 21-22 section 4<br>• Change in the schedule of blood draws and in the amount of blood collected on pp. 13-14 (section 1), pp.17-18 (table 2, section 7), p.43 (section 12.3)
4	29/04/2022	4	• New cohort of patients with chordoma included<br>• The centers to which Dr. Javier Martín and Dr. David da Silva Moura belong are modified<br>• Contact information: Address of the CRO updated<br>• Protocol signature page: GEIS president updated<br>• List of abbreviations updated<br>• 2.1: The name of this section is modified (for STS and bone sarcomas)<br>• 2.2: The section "Background and scientific rationale for chordomas" is added justifying the addition of the cohort of patients with chordoma<br>• 3.2: Objective response rate is changed to overall response rate<br>• 3.2: It is clarified that CTCAE 5.0 will be used in the second phase of the study (chordomas)<br>• 4.1: In the inclusion criteria, those for the new cohort of individuals with chordoma are added<br>• 4.2: In the exclusion criteria, those for the new cohort of individuals with chordoma are added

			• 4.4: The concept of pre-screening is eliminated as now, in the new cohort of patients with chordomas, only screening will be performed<br>• 6.1: New section added for the dosage form of palbociclib<br>• 7.1: Legends 1 and 12 below the test table are modified<br>• 7.3: CDKN2A mutation verification required for inclusion of patients with chordomas in phase 2 is added<br>• 7.3: More detail on baseline biopsy for translational study is included<br>• 7.3: The shipping address for tumor samples is changed<br>• 7.6: It is clarified that CTCAE 5.0 will be used for phase 2 of chordomas<br>• 11.2: “Objective response rate” is changed to “overall response rate” and it is clarified that CTCAE 5.0 will be used for phase 2 of chordomas<br>• 11.3: The sample size is redefined for the initial phase of STS and bone sarcomas, which is terminated. The sample size of the second phase, for chordomas only, is also added<br>• 11.5: It is clarified that CTCAE 5.0 will be used in phase 2 of chordomas<br>• 12.3: More detail on baseline biopsy is added for the translational substudy. Blood samples are specified as mandatory.<br>• 12.4.1: “Institute of Biomedicine of Seville (IBiS)” is replaced by “central translational laboratory”<br>• 12.4.2: Point 2): blood sample collections are correctly specified<br>• 12.4.2: The address for sending samples (CITIUS III) is updated<br>• 4.2: Exclusion criterion 15 is modified to refer to pneumonitis and ILD (chordoma cohort)<br>• 4.3: Pneumonitis/ILD is included in the reasons for permanent withdrawal<br>• 8.6: More specific surveillance for pneumonitis/ILD is included

CONTACT INFORMATION

Sponsor:

Grupo Español de Investigación en Sarcomas (GEIS)
Diego de León, 47
28006 Madrid, Spain
www.grupogeis.org

CRO assigned by the Sponsor as the main point of contact:

Sofpromed Investigación Clínica, SLU
Gran Vía Asima, 20, 3rd Floor, Office 23
07009 Palma de Mallorca, Spain
Tel: +34 648 414 261
Fax: +34 971 570 222
ensayos@sofpromed.com

LIST OF TABLES

Table 1: Dose modifications 27

Table 2: Schedule of activities 31

Table 3: Laboratory tests 35

Table 4: Response evaluation criteria according to Choi/RECIST 36

Table 5: Biological samples of the trial 49

PROTOCOL SIGNATURES PAGE

Sponsor: Grupo Español de Investigación en Sarcomas (GEIS)

Study title: Multicenter phase II trial of palbociclib in second line of advanced sarcomas with CDK4 overexpression

Sponsor's protocol code: GEIS-51

EudraCT Number: 2016-004039-19

Protocol version and date: 4 of 29/04/2022

I have read this protocol and agree to participate in this study by adhering to the protocol, regulatory requirements, Good Clinical Practice (GCP) guidelines, and the Declaration of Helsinki.

Sponsor:

……………………………..
Dr. Claudia Valverde
Grupo Español de Investigación en Sarcomas (GEIS)
President

Coordinating Investigators:

……………………………..
Dr. Javier Martín
Medical Oncologist
Hospital Universitario Fundación Jiménez Díaz

……………………………..
Dr. Roberto Díaz
Medical Oncologist
Hospital Universitari i Politècnic la Fe

Name of the Principal Investigator of the site:…………………………………………..

Name of the site:…………………………………………………………….……….……..

Signature of the Principal Investigator of the site:…………...…………………………

Date of signature:……………………………………………………………………..

TABLE OF CONTENTS

LIST OF TABLES **5**
1. SUMMARY **10**
2. INTRODUCTION **19**
2.1 Scientific background and rationale for STS and bone sarcomas 19
2.2 Scientific background and rationale for chordomas 20
2.3 Palbociclib 20
3. OBJECTIVES OF THE CLINICAL STUDY **21**
3.1 Main objective 21
3.2 Secondary objectives 21
4. PATIENT SELECTION **22**
4.1 Inclusion criteria 22
4.2 Exclusion criteria 23
4.3 Patient withdrawal criteria 24
4.4 Screen failures 25
5. TREATMENT **26**
5.1 Treatment administration 26
5.2 Interruptions and dose modifications 26
5.3 Treatment overdose 27
5.4 Concomitant medication 27
5.5 Pregnancy and lactation 28
6. MEDICATION MANAGEMENT **29**
6.1 Pharmaceutical form of palbociclib 29
6.2 Supply of study medication 29
6.3 Packaging, labelling and distribution 29
6.4 Storage in local pharmacies 29
6.5 Dispensation to patients 29
6.6 Drug accountability 29
6.7 Treatment adherence 29
6.8 Responsibilities of local pharmacies 29
7. PROCEDURES AND EVALUATIONS **31**
7.1 Schedule of activities 31
7.2 Patient information and consent 32
7.3 Eligibility and inclusion procedures 32
7.4 Clinical evaluations 34
7.5 Laboratory tests 34
7.6 Safety evaluations 35
7.7 Efficacy evaluations 36
7.8 Treatment control 37
7.9 Collection of biological samples 37
7.10 Follow-up 38
8. PHARMACOVIGILANCE **39**
8.1 Definitions 39
8.2 Recording and reporting of adverse events 39
8.3 Reporting of serious and unexpected adverse reactions 40

8.4 Other obligations of the Sponsor in terms of safety 41
8.5 Safety reports 41
8.6 Surveillance of specific adverse reactions 41
9. DATA MANAGEMENT 42
9.1 Electronic case report form (eCRF) 42
9.2 Custody and maintenance of documentation 42
10. MONITORING 44
11. STUDY DESIGN AND STATISTICS 45
11.1 Study design 45
11.2 Analytical variables 45
11.3 Sample size and statistical plan 45
11.4 Efficacy analysis 46
11.5 Safety analysis 46
12. TRANSLATIONAL SUBSTUDY 47
12.1 Rationale 47
12.2 Objectives of the translational study 48
12.3 Collection of biological samples 48
12.4 Methods and experiments 49
13. ETHICAL AND REGULATORY ASPECTS 51
13.1 Ethics committee 51
13.2 Competent authority 51
13.3 Ethical framework of the study 51
13.4 Patient privacy 51
13.5 Patient information sheet and informed consent 51
13.6 Insurance policy 52
14. TRIAL GOVERNANCE AND RESPONSIBILITIES 53
14.1 Trial Steering Committee (TSC) 53
14.2 Trial Management Group (TMG) 53
14.3 Responsibilities of the Sponsor 53
14.4 Responsibilities of the Principal Investigators 53
15. CONSIDERATIONS ON THE DEVELOPMENT OF THE STUDY 54
15.1 Inclusion of the study in clinical trial registries 54
15.2 Quality control 54
15.3 Definition of end of study 54
15.4 Criteria for suspension of the Sponsor 54
15.5 Publication of results 54
APPENDIX A: CREATININE CLEARANCE (CrCl) MEASUREMENTS 56
APPENDIX B: DECLARATION OF HELSINKI 57

LIST OF ABBREVIATIONS

ABP	Arterial Blood Pressure
AE	Adverse Event
ALT	Alanine aminotransferase
AST	Aspartate aminotransferase
BSA	Body Surface Area
CBR	Clinical Benefit Rate
CR	Complete Response
CRO	Contract Research Organization
CT scan	Computed Tomography
ECG	Electrocardiogram
ECOG	Eastern Cooperative Oncology Group
eCRF	Electronic Case Report Form
EORTC	European Organization for Research and Treatment of Cancer
EudraCT	European Union Drug Regulating Authorities Clinical Trials
FPFV	First Patient First Visit
GCP	Good Clinical Practice
GEIS	Grupo Español de Investigación en Sarcomas
HPF	High Power Field
ICH	International Conference on Harmonization
INR	International Normalized Ratio
ISG	Italian Sarcoma Group
L	Liter
LLN	Lower Limit of Normal
LPFV	Last Patient First Visit
LVEF	Left Ventricular Ejection Fraction
mg	Milligram
mg/m^2	Milligram per meter squared
mg/kg	Milligram per kilogram
µg	Microgram
mL	Milliliter
mL/min	Milliliter per minute
mm	Millimeter
msec	Millisecond
MUGA	Multi-gated Radionuclide Angiography
MVD	Microvessel Density
NCI CTCAE 4.0/5.0	National Cancer Institute Common Terminology for Coding of Adverse Events version 4.0/5.0
NMR	Nuclear Magnetic Resonance
ORR	Overall Response Rate
OS	Overall Survival
PD	Progressive Disease
PET	Positron Emission Tomography
PFS	Progression Free Survival
PI	Principal Investigator
PIS/IC	Patient Information Sheet/Informed Consent
PR	Partial Response
RECIST	Response Evaluation Criteria in Solid Tumors
SAE	Serious Adverse Event
SD	Stable Disease
SGOT	Serum Glutamic Oxaloacetic Transaminase
SGPT	Serum Glutamic Pyruvic Transaminase
STS	Soft Tissue Sarcoma
SUSAR	Suspected Unexpected Serious Adverse Reaction
TKI	Tyrosine Kinase Inhibitor
TMG	Trial Management Group
TSC	Trial Steering Committee
ULN	Upper Limit of Normal

1. SUMMARY

**Title of the trial**	Multicenter phase II trial of palbociclib in second line of advanced sarcomas with CDK4 overexpression
**Sponsor's protocol code**	GEIS-51
**EudraCT Number**	2016-004039-19
**Type of study**	National, multicenter, phase II, single-cohort, open-labe,l nonrandomized clinical trial with drugs.
**Coordinating Investigators**	Clinical trial:<br>• Dr. Javier Martín – Hospital Universitario Fundación Jiménez Díaz (Madrid)<br>• Dr. Roberto Díaz – Hospital Universitari i Politècnic La Fe (Valencia)<br><br>Translational substudy:<br>• Dr. Amancio Carnero – Hospital Universitario Virgen del Rocío (Seville)<br>• Dr. Marco Pérez – Hospital Universitario Virgen del Rocío (Seville)<br>• Dr. David da Silva Moura – Instituto de Investigación Sanitaria Fundación Jiménez Díaz (Madrid)
**Planned schedule**	**Cohort 1 (first phase) – STS and BS**<br><br>• Administrative approvals: 3 months<br>• First visit of first patient: Second trimester 2017<br>• Total recruitment period: 54 months<br>• Follow-up period: 12 months<br>• Estimated end date of first phase: Third quarter 2022<br>• Estimated date of analysis of results: Fourth quarter 2022<br><br>**Cohort 2 (second phase) – Chordomas**<br><br>• First visit of first patient: First quarter 2022<br>• Total recruitment period: 24 months<br>• Follow-up period: 12 months<br>• Estimated end date of second phase: First quarter 2025<br>• Estimated date of analysis of results: Second quarter 2025
**Estimated accrual rate**	2 cases per month
**Clinical objectives**	**Cohort 1 (first phase) and Cohort 2 (second phase)**<br><br>Main objective:

	• To determine the progression free survival (PFS) rate according to RECIST 1.1 at 6 months<br><br>Secondary objectives:<br><br>• To evaluate the overall response rate (ORR) according to RECIST 1.1 criteria<br>• To evaluate the response according to Choi criteria<br>• To evaluate the median PFS<br>• To evaluate the PFS rate at 3 months<br>• To evaluate the overall survival (OS)<br>• To evaluate the clinical benefit rate (CBR)<br>• To evaluate the safety profile according to CTCAE 4.0 (first phase; STS and BS) and 5.0 (second phase; chordomas)<br>• To evaluate the correlation of clinical data with molecular variables derived from the translational study
**Disease under study**	**Cohort 1 (first phase) – STS and BS**<br><br>Advanced soft tissue sarcomas and bone sarcomas<br><br>**Cohort 2 (second phase) – Chordomas**<br><br>Advanced chordomas
**Sample size**	**Cohort 1 (first phase) – STS and BS**<br><br>• Stage 1: 15 patients<br>• Stage 2: 6 patients<br>• Total: 21 patients<br><br>**Cohort 2 (second phase) – Chordomas**<br><br>• Total: 19 patients
**Treatment**	Palbociclib will be administered orally at a dose of 125 mg once daily for 21 consecutive days, followed by 7 days off to complete a 28-day cycle.<br><br>Treatment will continue until disease progression, development of unacceptable toxicity, protocol non-compliance, withdrawal of consent by the patient or by decision of the investigator.
**Drug information**	Name: Palbociclib (IBRANCE)<br>Presentation: Opaque hard gelatin capsules<br>Administration: Oral
**Inclusion Criteria**	**Cohort 1 (first phase) – STS and BS**<br><br>1) CDK4 overexpression (mRNA expression) and low or normal p16 expression (mRNA expression) measured in paraffin-embedded tumor samples at study entry.<br>2) ECOG 0-1 at the time of enrollment.<br>3) Diagnosis of soft tissue sarcoma or bone sarcoma (in both cases with metastasis or locally advanced inoperable).<br>4) Documented disease progression during the 6 months prior to study entry.

	5) Patients must have the following laboratory results:<br>• Absolute neutrophil count ≥ 1,500/mm³ (1.5 x 10^9/L);<br>• Platelets ≥ 100,000/mm³ (100 x 10^9/L);<br>• Hemoglobin ≥ 9 g/dL (90 g/L);<br>• Blood creatinine level ≤ 1.5 x ULN or estimated creatinine clearance ≥ 60 mL/min;<br>• Total level of blood bilirubin ≤ 1.5 x ULN (≤ 3.0 x ULN if Gilbert's disease);<br>• AST and/or ALT ≤ 3 x ULN (≤ 5.0 x ULN if liver metastasis is present);<br>• Alkaline phosphatase ≤ 2.5 x ULN (≤ 5.0 x ULN if bone or liver metastases are present);<br>6) Patients must have signed written consent to participate in the clinical study, and to provide at least two tumor blocks in paraffin for molecular analyses in the screening phase.<br>7) Biopsy at baseline if there are no archive tumor samples obtained in the 3 months prior to the start of treatment.<br>8) Patients must have received standard treatments for at least one, two or three lines for advanced disease.<br>9) Age between 18 and 80 years (both ages included).<br>10) Measurable disease according to RECIST 1.1 criteria.<br>11) All patients (men and women) of childbearing age must use an effective method of contraception throughout treatment with palbociclib and for at least 90 days after the last dose. Pregnancy must be ruled out by urine or blood test (negative pregnancy test) for inclusion in the study. Men should be advised to consider sperm preservation before starting treatment due to the risks of infertility.<br><br>**Cohort 2 (second phase) – Chordomas**<br><br>1) *CDKN2A* gene mutation.<br>2) ECOG 0-1 at the time of enrollment.<br>3) Diagnosis of chordoma (metastatic or locally advanced inoperable) centrally confirmed.<br>4) Disease progression according to RECIST 1.1, within one year prior to inclusion, to previous treatment (surgery, radiotherapy, or systemic treatment).<br>5) Patients are not candidates for surgical salvage or radiotherapy at the time of inclusion.<br>6) Patients must have the following laboratory results:<br>• Absolute neutrophil count ≥ 1,500/mm³ (1.5 x 10^9/L);<br>• Platelets ≥ 100,000/mm³ (100 x 10^9/L);<br>• Hemoglobin ≥ 9 g/dL (90 g/L);<br>• Blood creatinine level ≤ 1.5 x ULN or estimated creatinine clearance ≥ 60 mL/min;<br>• Total level of blood bilirubin ≤ 1.5 x ULN (≤ 3.0 x ULN if Gilbert's disease);<br>• AST and/or ALT ≤ 3 x ULN (≤ 5.0 x ULN if liver metastasis is present);<br>• Alkaline phosphatase ≤ 2.5 x ULN (≤ 5.0 x ULN if bone or liver metastases are present);<br>7) Patients must have signed written consent to participate in the clinical study, and to provide at least two tumor blocks in paraffin for molecular analyses in the screening phase.<br>8) Biopsy at baseline if there are no archive tumor samples obtained in the 3 months prior to the start of treatment. If there are tumor samples in this period, there must have been no subsequent treatments.<br>9) Patients may have received up to 3 lines of systemic treatment previously.<br>10) Age between 18 and 80 years (both ages included).<br>11) Measurable disease according to RECIST 1.1 criteria.<br>12) All patients (men and women) of childbearing age must use an effective method of contraception throughout treatment with palbociclib and for at least

	90 days after the last dose. Pregnancy must be ruled out by urine or blood test (negative pregnancy test) for inclusion in the study. Men should be advised to consider sperm preservation before starting treatment due to the risks of infertility.
**Exclusion Criteria**	**Cohort 1 (first phase) – STS and BS**<br><br>1) Prior treatment with any anti-CDK4 or immune checkpoint inhibitors.<br>2) Diagnosis of Ewing's sarcoma or rhabdomyosarcoma.<br>3) Diagnosis of well-differentiated/dedifferentiated liposarcoma.<br>4) Patients irradiated on the only available target lesion.<br>5) Patients who have received more than three lines for advanced disease.<br>6) History of other neoplastic disease except for adequately treated basal cell carcinoma or cervical cancer in situ.<br>7) Severe cardiovascular disease (NYHA >=2).<br>8) Toxicity grade 3 or higher according to CTCAE 4.0 if, in the opinion of the investigator, it may significantly interfere with the toxicity of the drug under study.<br>9) Patients who have not recovered from previous toxicity up to CTCAE grade 1 due to previous antineoplastic treatment with chemotherapy, radiotherapy, or biological therapy (including monoclonal antibodies).<br>10) Patients who have not recovered from minor or major surgery or who have had major surgery in the 4 weeks prior to the start of study treatment.<br>11) Metastasis in the central nervous system.<br>12) Patients who are pregnant or breastfeeding, or who expect to conceive children during the treatment period.<br>13) Foods or drugs known to be CYP3A4 inhibitors/inducers; CYP3A4 substrates with narrow therapeutic ranges or known to prolong the QTc interval.<br>14) Major surgery, chemotherapy, radiotherapy, any investigational agent, or other antineoplastic therapy within 4 weeks prior to enrollment. Patients who have received previous radiotherapy ≥25% of the bone marrow are ineligible, regardless of when it was received.<br>15) QTc > 480 ms; personal or family history of long or short QT syndrome, Brugada syndrome or known history of QTc prolongation, or Torsades de Pointes (TdP).<br>16) Any of the following situations within 6 months prior to study drug administration: myocardial infarction, severe/unstable angina, current cardiac dysrhythmias of Grade ≥ 2 NCI-CTCAE version 4.0, atrial fibrillation of any grade, coronary/peripheral artery pacemaker implantation, symptomatic congestive heart failure, cerebrovascular accident including transient ischemic attack, or symptomatic pulmonary embolism.<br>17) Known hypersensitivity to any PD 0332991 or excipients.<br>18) Active or recent suicidal intent or behavior.<br><br>**Cohort 2 (second phase) – Chordomas**<br><br>1) Prior treatment with any anti-CDK4 or immune checkpoint inhibitors.<br>2) Diagnosis other than chordoma at the central review.<br>3) Patients irradiated on the only available target lesion.<br>4) Patients who have received more than three lines for advanced disease.<br>5) History of other neoplastic disease except for adequately treated basal cell carcinoma or cervical cancer in situ. This criterion will be assessed individually with the research team.<br>6) Severe cardiovascular disease (NYHA >= 2).<br>7) Toxicity grade 3 or higher according to CTCAE 5.0 if, in the opinion of the investigator, it may significantly interfere with the toxicity of the drug under study.

	8) Patients who have not recovered from previous toxicity up to CTCAE grade 1 due to previous antineoplastic treatment with chemotherapy, radiotherapy, or biological therapy (including monoclonal antibodies).<br>9) Patients who have not recovered from minor or major surgery or who have had major surgery in the 4 weeks prior to the start of study treatment.<br>10) Metastasis in the central nervous system.<br>11) Patients who are pregnant or breastfeeding, or who expect to conceive children during the treatment period.<br>12) Foods or drugs known to be CYP3A4 inhibitors/inducers; CYP3A4 substrates with narrow therapeutic ranges or known to prolong the QTc interval.<br>13) Major surgery, chemotherapy, radiotherapy, any investigational agent, or other antineoplastic therapy within 4 weeks prior to enrollment. Patients who have received previous radiotherapy ≥25% of bone marrow are ineligible, regardless of when it was received.<br>14) QTc > 480 ms; personal or family history of long or short QT syndrome, Brugada syndrome or known history of QTc prolongation, or Torsades de Pointes (TdP).<br>15) Any of the following situations within 6 months prior to study drug administration: myocardial infarction, severe/unstable angina, current cardiac dysrhythmias of Grade ≥ 2 NCI-CTCAE version 5.0, atrial fibrillation of any grade, coronary/peripheral artery pacemaker implantation, symptomatic congestive heart failure, cerebrovascular accident including transient ischemic attack, symptomatic pulmonary embolism, or severe interstitial lung disease (ILD)/pneumonitis.<br>16) Known hypersensitivity to any PD 0332991 or excipients.<br>17) Active or recent suicidal intent or behavior.
**Statistical design**	**Cohort 1 (first phase)**<br><br>According to the initial design for the first phase of STS and BS (Simon's two-stage), for an alpha error of 0.1 and a power (beta error) of 0.90, H0 14% (cut-off for active drugs, progression free survival rate at 6 months according to EORTC experience) and H1 35% of progression free survival rate at 6 months were estimated. In the first stage, 15 patients were to be recruited of whom at least 3 had to have a progression free survival greater than 6 months. If this occurred, then a second stage would continue with further 23 patients to reach a total of 38 evaluable patients. If at least 9 patients had 6-month PFS or longer, further investigation of the drug would be warranted.<br><br>The design of the first phase has been amended preserving, to the greatest extent possible, the differences between H0 and H1, due to low recruitment. The new approach is a Simon's two-stage Minimax design (type 1 and type 2 errors at 10%), H0 15% and H1 40%. If 2 or fewer successes are observed in the first 15 patients recruited, the trial will be stopped in this cohort with the conclusion that the investigational drug should not be further investigated in this subgroup of patients. In another case (3 or more successes in the first 15 patients recruited, PFS greater than 6 months) patient recruitment will continue up to 21 evaluable patients. If 6 or more successes are observed in these 21 patients, it will be concluded that the results of this trial deserve further investigation.<br><br>**Cohort 2 (second phase)**<br><br>For the chordoma cohort (second phase) the main endpoint is the proportion of patients with progression free survival (PFS) at 6 months. Since the mechanism of action is based on a reduction of viability in relation to G1 cell cycle arrest rather than apoptosis, it seems more reasonable to keep this endpoint.

<table>
<tr><td></td><td>The first prospective study in chordoma with a systemic drug (9-nitro-camptothecin) demonstrated a 33% PFS rate at 6 months[32]. Stacchiotti reported a PFS rate of 65% at 6 months[33], while Schueltze obtained a PFS rate of 54% with dasatinib at 6 months[34].

A Simon's two-stage Minimax design is considered, with 10% of type 1 and type 2 errors. Success at 30% or less will be considered unacceptable and would not involve further investigation (null hypothesis). Thus, the value of H0 will be 30%. Success in 60% of cases or more will be considered an acceptable outcome (H1, alternative hypothesis, 60%).

A total of 9 eligible and treated patients will be included in the first stage of this cohort. If 2 or less successes are observed, then the trial will be stopped in this cohort with the conclusion that the investigational drug should not be investigated further in chordoma. In another case (3 successes or more), recruitment will continue until 19 evaluable patients have entered the study. If 9 or more patients are successful among these 19 patients, it will be concluded that the results of this trial deserve further investigation.</td></tr>
<tr><td>Clinical trial endpoints</td><td>Cohort 1 (first phase) and Cohort 2 (second phase)

Main endpoint

• Efficacy measured by progression free survival (PFS) rate at 6 months, assessed by RECIST 1.1 criteria.

Secondary endpoints

• Efficacy measured by overall response rate (ORR) (complete response [CR] and partial response [PR]), assessed during RECIST 1.1 criteria. The evaluation criteria will be based on identification of the target lesions at baseline and follow-up of these lesions until tumor progression.
• Efficacy measured by response according to Choi criteria. The evaluation criteria will be based on identification of target lesions at baseline and follow-up until tumor progression.
• Efficacy measured by the median PFS.
• Efficacy measured by the PFS rate at 3 months.
• Overall survival (OS) measured from the date of initiation of treatment with palbociclib to the date of death, regardless of cause.
• Clinical benefit rate (CBR). Patients who have shown complete response, partial response or disease stabilization for 6 months or more, showing signs of clinical improvement, will be considered to have experienced clinical benefit.
• Safety profile of palbociclib, by evaluation of adverse events (type, incidence, severity, time of occurrence, related causes) observed on physical examinations and laboratory tests. Toxicity will be analyzed and tabulated using NCI-CTCAE 4.0 (first phase; STS and OS) and 5.0 (second phase; chordomas).</td></tr>
<tr><td>Translational substudy objectives</td><td>1.- Assess the expression of mRNA and CDK4 protein (and $p16^{ink4a}$) in tumor samples from patients diagnosed with sarcoma (blood and paraffin block), to identify the most reliable and effective method to select CDK4 and/or $p16^{ink4a}$ as a predictive biomarker of palbociclib efficacy.

2.- Evaluate the predictive and/or prognostic value, at mRNA and protein level, of p53, $p21^{CIP1}$, $p27^{KIP1}$, Rb, FoxM1 and cyclin D1 as potential biomarkers of palbociclib efficacy, in blood samples and in paraffin blocks (pre- and post-treatment).

3.- Analyze other senescence markers (combination of Ki67 negative with high density (>5 per nucleus) of γH2A.X foci) by immunohistochemistry of paraffin blocks (pre- and</td></tr>
</table>

	post-treatment). Pre- and post-treatment samples from each patient will be compared to determine senescence induced by palbociclib treatment.<br><br>4.- Correlate biomarkers predictive of palbociclib efficacy with clinical outcomes such as PFS, OS, and clinical benefit rates (complete response, partial response, and stable disease).<br><br>5.- Evaluate the effect of palbociclib in modulating the immune response in paraffin block samples from patients undergoing chemotherapy. The evaluation will be performed on pre- and post-treatment samples from each patient.

Procedures and evaluations

Protocol section	Stages >>	PRE-TREAT.	TREATMENT												END TREAT.[2] (+/- 7 days)	FOLLOW-UP (+/- 7 days)
		**Screening[1] (≤ 28 days from start of treatment)**	**C1 D1**	**C1 D14**	**C2 D1**	**C2 D14**	**C3 D1**	**C4 D1**	**C5 D1**	**C6 D1**	**C7 D1**	**C8 D1**	**C9 D1**	**C10 D1 … Cn D1**		
**7.2**	**Patient information and consent**															
	Information to the patient	X (before screening)														
	Informed consent	X (before screening)														
**7.3**	**Eligibility and inclusion procedures**															
	CDK4 overexpression analysis	X														
	Eligibility confirmation	X														
	Patient recruitment	X														
**7.4**	**Clinical evaluations**															
	Demographics and medical history	X														
	ECOG	X	X	X	X	X	X	X	X	X	X	X	X	X	X	
**7.5**	**Laboratory tests[3] (+/- 3 days)**															
	Biochemistry[4]	X	X	X	X	X	X	X	X	X	X	X	X	X	X	
	Hematology[5]	X	X	X	X	X	X	X	X	X	X	X	X	X	X	
	Coagulation tests	X														
	Pregnancy test[6]	X						X			X			X[6]		
**7.6**	**Safety evaluations**															
	Physical examination[7]	X	X	X	X	X	X	X	X	X	X	X	X	X	X	
	Vital signs	X	X	X	X	X	X	X	X	X	X	X	X	X	X	
	Adverse events		Throughout the course of treatment													
	Concomitant medication	X	Throughout the course of treatment													
	ECG[8]	X														
	LVEF	X													X	
**7.7**	**Efficacy evaluations**															
	Radiological evaluation[9]	X	Every 8 weeks (irrespective of cycle delays)												X	
	Additional imaging tests[10]	X	Every 8 weeks													
	Central radiological review														X	
**7.8**	**Treatment control**															
	Medication dispensing[11]		X		X		X	X	X	X	X	X	X	X		
	Intake of medication		Palbociclib 125 mg once daily for 3 weeks followed by 1 week off (28-day cycles)													
	Treatment adherence		Every 4 weeks													
**7.9**	**Collection of biological samples**															
	Tumor block collection[12]	X														
	Blood collection[13]	X			X	Every 8 weeks (after each radiological evaluation)									X	
**7.10**	**Follow-up**															
	Follow-up visits															Every 3 months until death

1. Documentation of the *CDKN2A* mutation (chordoma cohort) and a central diagnostic confirmation (STS, BS, and chordoma cohorts) is required for participation in the trial. For STS and BS cohort, a pre-screening test before screening is established, for the first CDK4 expression analysis done on already existing tumor block.
2. End-of-treatment evaluation must be done at the end of medication administration (within 4 weeks)
3. Baseline laboratory testing should be done within 7 days prior to initiation of treatment
4. Biochemistry: ALT, AST, total bilirubin, creatinine, alkaline phosphatase, glucose, albumin, total protein, LDH, chloride, phosphorus, sodium, potassium, calcium
5. Hematology: hematocrit, hemoglobin, leukocytes, red blood cells, neutrophils, platelets, RDW
6. Women of reproductive age. The pregnancy test should be done before starting treatment (within 7 days prior to inclusion) and then every 3 cycles until the end of treatment
7. In addition to other physical examinations (see section 7.6), weight and height are required at baseline, and height is not required on day 1 of each cycle
8. ECG within 14 days prior to enrollment
9. Preferably CT scan
10. FDG-PET, MRI diffusion, bone scan (if indicated)
11. Medication is dispensed to the patient on the first day of each cycle
12. A biopsy in the baseline period (within 28 days prior to the start of treatment) will be necessary if there is no archive tumor specimen obtained in the 3 months prior (no systemic treatment must be present in this period) to the initiation of treatment
13. Blood collections: (1) Within 72 hours pre-treatment, (2) at the end of the first cycle (day 28), (3) after each radiological evaluation

2. INTRODUCTION

2.1 Scientific background and rationale for STS and bone sarcomas

Sarcomas are rare malignant tumors of mesenchymal origin that affect bones, soft tissues (STS) and viscera. They are one of the most devastating solid tumors in children and adults, and their rarity and heterogeneity (more than 60 subtypes) hinders the development of new effective therapies.

Today, the incidence of STS is around 60 new cases per year per million people, with 20% of them being metastatic at the time of diagnosis, and the other 40% having distant metastases within the following 5 years. In metastatic cases, complete responses to chemotherapy are very rare and the median survival is about 1.5 years[1].

Schemes with anthracyclines and ifosfamide at adequate doses are the most used as first line, especially for whom further lesion shrinkage may lead to improved long-term outcomes[2,3]. However, the most optimistic expectation of the combination in terms of response rate and progression free survival is less than 30%, and less than 8 months respectively, according to phase III trials in advanced disease[4]. These results have hardly improved in the last two decades despite some evidence of dose-response relationship for both drugs. Hence, there is a conviction that there is a ceiling in terms of efficacy for the most active chemotherapy in soft tissue sarcomas (STS).

Therefore, new treatments for sarcomas are desperately needed, especially for patients with advanced or metastatic disease, for whom no useful curative intent can be offered.

Histological grade is a relevant prognostic factor in sarcoma patients and a significant correlation with response has been identified: the higher the grade, the greater the likelihood of response[5]. The grading system in sarcoma is based on the percentage of necrosis, differentiation, and the mitotic index. The latter to play an important role in the *cinsarc* gene signature, as the 67 genes included in these signatures are involved in mitosis and in the control of chromosome integrity[6]. Based on this, previous information suggests that focusing on cell cycle might be relevant in sarcomas.

CDK4 and CDK6, which are activated by D-type cyclins, integrate signals from mitogens and/or anti-proliferative factors to regulate the initiation of phase G1 by phosphorylating the tumor suppressor pRb[7]. This leads to inactivation of pRb and release of the transcription factor E2F, whose activity is necessary for DNA synthesis and cell cycle progression. pRb phosphorylation is then maintained independently of the D-type cyclins through the transcription dependent on E2F of cyclin E, this leading to activation of CDK2 and further phosphorylation of pRb[8]. In addition, cyclin D-CDK4/6 complexes perform a second, non-catalytic function in the G1 phase of progression by isolating the Cip/Kip CDK inhibitors, p21 and p27, thus facilitating CDK29 activation[9]. Progression through G1 is also limited by the abundance of CDK inhibitors (CKIs) such as the proteins p16INK4, which block the cyclin D binding site on CDK4/6, and p27KIP1, which has broad specificity for cyclin-CDK10 complexes[10]. If the requirements for the G1 checkpoint transition are not met, the cell exits the cell cycle and enters the quiescent state (G0) or, under some circumstances, senescence (permanent cell cycle arrest). CDK4 and CDK6 perform different overlapping functions[11].

Aberrant cell cycle regulation is a characteristic feature of cancer, and multiple mechanisms contribute to G1-to-S checkpoint dysregulation[12]. These mechanisms include amplification or mutation of CDK4 and CDK6 genes, amplification of genes encoding D-type cyclins, and deletion or silencing of the CDKN2A/B gene, which encodes the inhibitors INK4, p16ink4a and p15ink4b[13]. Additionally, aberrant expression of growth factors or growth factor receptors can activate cells to produce cyclin D1 in an autocrine manner. These pathways can also be constitutively activated due to overexpression of downstream signaling molecules that control cyclin D1 expression, such as Ras proteins, which are frequently overexpressed in cancer, and are associated with poor prognosis. Cell cycle dysregulation is crucial for several oncogenic transformation processes, suggesting that many cancer cells depend on high CDK4/6 activity[14,15]. In contrast, normal development of most tissues can take place in the absence of cyclins; cyclin DCDK4/6 complexes[16]. Thus, CDK4/6 activity appears to represent a promising therapeutic target for cancer treatment[17]. In the context of sarcoma, aberrant expression of Rb pathway proteins is common, suggesting that these proteins may be involved in oncogenesis. Indeed, CDK4 overexpression at the protein level was detected in 92% of sarcomas in a series of 152 cases[18].

2.2 Scientific background and rationale for chordomas

Chordoma tumor cells frequently exhibit aberrant expression of *CDK4/CDK6* due to the loss of p16, a protein encoded by the *CDKN2A* gene. The latter is considered a tumor suppressor gene as p16 is an inhibitor of *CDK4/CDK6* and, therefore, a crucial regulator of cell growth and proliferation. Loss of *CDKN2A* is related to aggressive behavior of several tumors[19].

CDK4 overexpression at mRNA level was observed in 7 out of 10 fresh primary chordoma specimens, one showed intermediate expression and the other two, low expression. No patient showed p16 expression. In other words, 80% of patients with chordoma would fit the inclusion criteria of the study. When considering protein expression, 97.7% of chordomas (72 out of 85) expressed CDK4. Of the latter, 50.6% were classified as having elevated protein expression. The median CDK4 expression was higher in metastatic specimens than in primary chordomas[20].

In chordoma cell lines, widespread activation of CDK4/6 and Rb signaling pathways was detected, while all cells exhibited loss of *CDKN2A* and p16. This was identified in a chordoma tissue bank of 43 patients and in 8 established chordoma cell lines. In addition, palbociclib effectively inhibited tumor cell growth in vitro, demonstrated by immunohistochemistry of the CDK4/p1621 system[21]. The in vitro effect of palbociclib on chordoma cell lines can be quantified by the decrease in pRb, with significant cell cycle arrest in G1, reduction in the fraction of cells in S phase, and decrease in cell viability in a dose-dependent manner. Increased apoptosis was not detected[20].

In summary, despite the existence of substantial genomic heterogeneity in chordoma, CDK4/p16 alteration is a common denominator. The new cohort added in our study could add value by exploring, at protein and mRNA levels, the CDK4/CDKN2A-p16 pairing in the reference samples, with the aim of finding out whether palbociclib efficacy may be related to CDK4/CDKN2A-p16 expression.

2.3 Palbociclib

Palbociclib (IBRANCE) is an oral inhibitor of cyclin-dependent kinases (CDKs) 4 and 6. CDKs 4 and 6 are key regulators of the cell cycle that trigger cell progression.

Palbociclib (PD0332991) is the first highly selective CDK4/6 inhibitor that has been tested and approved in humans in combination with letrozole for the treatment of postmenopausal women with advanced breast cancer, —estrogen receptor (ER) positive, and human epidermal growth factor receptor 2 (HER2) negative— as initial endocrine therapy for metastatic disease. Palbociclib shows a median inhibitory concentration (IC50) of 10-15 nms. for CDK4/6, compared to 0.5 µM for CDK2[22,23]. In fact, palbociclib has recently been tested in liposarcoma with elevated CDK4 expression[24,25]. Preclinical studies have shown that PD0332991 induces G1 in pRb-positive cell lines, and suppresses the growth of several xenografts[22,26,27]. In different cancer models, treatment with PD0332991 not only exerts a cytostatic effect, but also induces senescence or apoptotic cell death of tumor cells[28]. The only known mechanism of resistance to CDK4/6 inhibition is loss of pRb function[28,29]. Nevertheless, other mechanisms have been proposed, such as loss of p16ink4a, overexpression of cyclin D1 of elevated CDK2 expression[27,30].

The coordinating investigators of the GEIS-51 trial extensively explored CDK4/6 inhibition with PD0332991 in a panel of 10 low-passage sarcoma cell lines generated directly from patients, and in 3 additional commercial sarcoma cell lines, as well as in xenograft models. The most relevant findings were that palbociclib induces senescence in these cell lines, and that the response of these cell lines correlated with CDK4 RNA levels (above the median, it indicated better tumor control), always in the context of normal or low p16 expression[31].

3. OBJECTIVES OF THE CLINICAL STUDY

3.1 Main objective

- To determine the progression free survival (PFS) rate according to RECIST 1.1 at 6 months

3.2 Secondary objectives

- To evaluate the overall response rate (ORR) according to RECIST 1.1 criteria
- To evaluate the response according to Choi criteria
- To evaluate the median PFS
- To evaluate the PFS rate at 3 months
- To evaluate the overall survival (OS)
- To evaluate the clinical benefit rate (CBR)
- To evaluate the safety profile according to CTCAE 4.0 (first phase; STS and BS) and 5.0 (second phase; chordomas)
- To evaluate the correlation of clinical data with molecular variables derived from the translational study

4. PATIENT SELECTION

4.1 Inclusion criteria

Cohort 1 (first phase) – STS and BS

1) CDK4 overexpression (mRNA expression) and low or normal p16 expression (mRNA expression) measured in paraffin tumor samples at study entry.
2) ECOG 0-1 at the moment of enrollment.
3) Diagnosis of soft tissue sarcoma or bone sarcoma (in both cases with metastasis or locally advanced inoperable).
4) Documented disease progression during the 6 months prior to study entry.
5) Patients must have the following laboratory results:
    - Absolute neutrophil count ≥ 1,500/mm^3 (1.5 x 10^9/L);
    - Platelets ≥ 100,000/mm^3 (100 x 10^9/L);
    - Hemoglobin ≥ 9 g/dL (90 g/L);
    - Blood creatinine level ≤ 1.5 x ULN or estimated creatinine clearance ≥ 60 mL/min;
    - Total level of blood bilirubin ≤ 1.5 x ULN (≤ 3.0 x ULN if Gilbert’s disease);
    - AST and/or ALT ≤ 3 x ULN (≤ 5.0 x ULN if liver metastasis is present);
    - Alkaline phosphatase ≤ 2.5 x ULN (≤ 5.0 x ULN if bone or liver metastases are present);
6) Patients must have signed written consent to participate in the clinical study, and to provide at least two tumor blocks in paraffin for molecular analyses in the screening phase.
7) Biopsy at baseline if there are no archive tumor samples obtained in the 3 months prior to the start of treatment.
8) Patients must have received standard treatments for at least one, two or three lines for advanced disease.
9) Age between 18 and 80 years (both ages included).
10) Measurable disease according to RECIST 1.1 criteria.
11) All patients (men and women) of childbearing age must use an effective method of contraception throughout treatment with palbociclib and for at least 90 days after the last dose. Pregnancy must be ruled out by urine or blood test (negative pregnancy test) for inclusion in the study. Men should be advised to consider sperm preservation before starting treatment due to the risks of infertility.

Cohort 2 (second phase) – Chordomas

1) *CDKN2A* gene mutation.
2) ECOG 0-1 at the moment of enrollment.
3) Diagnosis of chordoma (metastatic or locally advanced inoperable) centrally confirmed.
4) Disease progression according to RECIST1.1 criteria, within one year prior to inclusion, to previous treatment (surgery, radiotherapy or systemic treatment).
5) Patients are not candidates for surgical salvage or radiotherapy at the moment of enrollment.
6) Patients must have the following laboratory results:
    - Absolute neutrophil count ≥ 1,500/mm^3 (1.5 x 10^9/L);
    - Platelets ≥ 100,000/mm^3 (100 x 10^9/L);
    - Hemoglobin ≥ 9 g/dL (90 g/L);
    - Blood creatinine level ≤ 1.5 x ULN or estimated creatinine clearance ≥ 60 mL/min;
    - Total level of blood bilirubin ≤ 1.5 x ULN (≤ 3.0 x ULN if Gilbert’s disease);
    - AST and/or ALT ≤ 3 x ULN (≤ 5.0 x ULN if liver metastases are present);
    - Alkaline phosphatase ≤ 2.5 x ULN (≤ 5.0 x ULN if bone or liver metastases are present);
7) Patients must have signed written consent to participate in the clinical trial, and to provide the tumor blocks in paraffin for molecular analyses in the screening phase.
8) Biopsy at baseline if there are no archive tumor samples obtained in the 3 months prior to the start of treatment. If there are tumor samples in this period, there should have been no subsequent treatments.
9) Patients must have received up to 3 lines of systemic treatment previously.
10) Age between 18 and 80 years (both ages included).
11) Measurable disease according to RECIST 1.1 criteria.
12) All patients (men and women) of childbearing age must use an effective method of contraception throughout treatment with palbociclib and for at least 90 days after the last dose. Pregnancy must be

ruled out by urine or blood test (negative pregnancy test) for inclusion in the study. Men should be advised to consider sperm preservation before starting treatment due to risks of infertility.

4.2 Exclusion criteria

Cohort 1 (first phase) – STS and BS

1) Prior treatment with any anti-CDK4 or immune checkpoint inhibitors.
2) Diagnosis of Ewing's sarcoma or rhabdomyosarcoma.
3) Diagnosis of well-differentiated/dedifferentiated liposarcoma.
4) Patients irradiated on the only available target lesion.
5) Patients who have received more than three lines for advanced disease.
6) History of other neoplastic disease except for adequately treated basal cell carcinoma or cervical cancer in situ.
7) Severe cardiovascular disease (NYHA >= 2).
8) Toxicity grade 3 or higher according to CTCAE 4.0 if, in the opinion of the investigator, it may significantly interfere with the toxicity of the drug under study.
9) Patients who have not recovered from previous toxicity up to CTCAE grade 1 due to previous antineoplastic treatment with chemotherapy, radiotherapy, or biological therapy (including monoclonal antibodies).
10) Patients who have not recovered from minor or major surgery or who have had major surgery in the 4 weeks prior to the start of study treatment.
11) Metastasis in the central nervous system.
12) Patients who are pregnant or breastfeeding, or who expect to conceive children during the treatment period.
13) Foods or drugs known to be CYP3A4 inhibitors/inducers; CYP3A4 substrates with narrow therapeutic ranges or known to prolong the QTc interval.
14) Major surgery, chemotherapy, radiotherapy, any investigational agent, or other antineoplastic therapy within 4 weeks prior to enrollment. Patients who have received previous radiotherapy ≥25% of the bone marrow are ineligible, regardless of when it was received.
15) QTc > 480 ms; personal or family history of long or short QT syndrome, Brugada syndrome or known history of QTc prolongation, or Torsades de Pointes (TdP).
16) Any of the following situations within 6 months prior to study drug administration: myocardial infarction, severe/unstable angina, current cardiac dysrhythmias of Grade ≥ 2 NCI-CTCAE version 4.0, atrial fibrillation of any grade, coronary/peripheral artery pacemaker implantation, symptomatic congestive heart failure, cerebrovascular accident including transient ischemic attack, or symptomatic pulmonary embolism.
17) Known hypersensitivity to any PD 0332991 or excipients.
18) Active or recent suicidal intent or behavior.

Cohort 2 (second phase) – Chordomas

1) Previous treatment with any anti-CDK4 or immune checkpoint inhibitors.
2) Diagnosis other than chordoma at central review.
3) Patients irradiated in the only available target lesion.
4) Patients who have received more than three lines for advanced disease.
5) History of other neoplastic disease except for adequately treated basal cell carcinoma or cervical cancer in situ. This criterion will be assessed individually with the research team.
6) Severe cardiovascular disease (NYHA >= 2).
7) Toxicity grade 3 or higher according to CTCAE 5.0 if, in the opinion of the investigator, it may significantly interfere with the toxicity of the drug under study.
8) Patients who have not recovered from previous toxicity up to CTCAE grade 1 due to previous antineoplastic treatment with chemotherapy, radiotherapy, or biological therapy (including monoclonal antibodies).
9) Patients who have not recovered from minor or major surgery or who have had major surgery in the 4 weeks prior to the start of study treatment.
10) Metastasis in the central nervous system.
11) Patients who are pregnant or breastfeeding, or who expect to conceive children during the treatment period.

12) Foods or drugs known to be CYP3A4 inhibitors/inducers; CYP3A4 substrates with narrow therapeutic ranges or known to prolong the QTc interval.
13) Major surgery, chemotherapy, radiotherapy, any investigational agent, or other antineoplastic therapy within 4 weeks prior to inclusion. Patients who have received previous radiotherapy ≥25% of bone marrow are ineligible, regardless of when it was received.
14) QTc > 480 ms; personal or family history of long or short QT syndrome, Brugada syndrome or known history of QTc prolongation, or Torsades de Pointes (TdP).
15) Any of the following situations within 6 months prior to study drug administration: myocardial infarction, severe/unstable angina, current cardiac dysrhythmias of Grade ≥ 2 NCI-CTCAE version 5.0, atrial fibrillation of any grade, coronary/peripheral artery pacemaker implantation, symptomatic congestive heart failure, cerebrovascular accident including transient ischemic attack, symptomatic pulmonary embolism, or severe interstitial lung disease (ILD)/pneumonitis.
16) Known hypersensitivity to any PD 0332991 or excipients.
17) Active or recent suicidal intent or behavior.

4.3 Patient withdrawal criteria

Permanent treatment discontinuation

Patients will receive the investigational medicinal product until any of the following events occur:

- Disease progression according to RECIST 1.1 criteria
- Unacceptable toxicity or an adverse event that, in the investigator's judgment, makes the administration of the study drug an unacceptable risk
- The patient has severe interstitial lung disease (ILD)/pneumonitis
- The investigator or Sponsor considers that the patient does not meet the requirements of the protocol
- A delay in treatment administration longer than 3 weeks
- The study is closed or terminated
- The patient withdraws consent to participate in the study

The reason for discontinuation of treatment should be clearly recorded in the eCRF.

A temporary discontinuation of study medication due to an adverse event is not considered a permanent discontinuation of the investigational product.

Withdrawal from the study

Patients will be encouraged to complete the study. However, they may voluntarily withdraw from the study at any time. The investigators may also withdraw, at their discretion, the patient from the study at any time.

Reasons for early withdrawal from the study must be recorded in the eCRF. These include:

- Closure/termination of the study
- Loss of patient follow-up
- Investigator's decision
- Patient's withdrawal of consent
- Major protocol violations
- Death

The date of withdrawal from the study, together with the reason for withdrawal, shall be recorded in the eCRF. In the case of death, a death certificate should be obtained as soon as possible, with the cause of death evaluated and documented.

Patients withdrawn from the trial for any reason cannot re-enter the trial.

4.4 Screen failures

A patient is considered a screen failure if he or she signs the informed consent for the trial but is withdrawn before inclusion in the study. All potential patients who go through the screening phase in this study (including screen failures) will be recorded in a list of screenings, even if some of them are not included in the eCRF. Reasons for exclusion will be recorded for candidates who did not enter the study.

5. TREATMENT

5.1 Treatment administration

Study treatment can only be initiated if the patient has signed the informed consent and the center has properly completed the enrollment process, for which the submission of two representative blocks of the tumor in paraffin from a biopsy obtained within 3 months prior to enrollment is mandatory.

The dose of IBRANCE for the trial is 125 mg taken orally once daily for 21 days, followed by 7 days off, in 28-day cycles. IBRANCE should be taken with food. Patients should take their dose at approximately the same time each day. The capsules should be swallowed whole. Capsules should not be taken if they are broken or not intact. If a patient vomits or misses a dose, an additional dose should not be taken on the same day. The next prescribed dose should be taken at the usual time.

Palbociclib should be started within seven days of the inclusion date (longer delays should be discussed with the study CRO).

5.2 Interruptions and dose modifications

Management of some adverse events may require temporary dose interruptions or delays and/or dose reductions, or permanent discontinuation. Recommended dose modifications of IBRANCE are based on individual safety and tolerability.

Complete blood counts should be monitored prior to initiation of IBRANCE treatment and at the beginning of each cycle, as well as on day 14 of the first two cycles, as clinically indicated.

**Recommended dose modifications for adverse events**	
Dose level	***Dose***
Recommended initial dose	125 mg/day
First dose reduction	100 mg/day
Second dose reduction	75 mg/day*

**If a dose reduction below 75 mg/day is required, treatment should be discontinued.*

Dose modification instructions for hematologic and non-hematologic toxicities are detailed below:

**Hematologic toxicities**	
CTCAE Grade 4.0	***Dose modifications***
Grade 1 or 2	No dose adjustment required.
Grade 3	Day 1 of cycle:<br>Discontinue IBRANCE, repeat full blood count monitoring in 1 week. When recovered to Grade ≤2, start next cycle at the *same dose*.<br><br>Day 14 of first 2 cycles:<br>Continue IBRANCE at current dose to complete the cycle. Repeat complete blood count on Day 21.<br><br>Consider dose reduction in cases of prolonged recovery (>1 week) of Grade 3 neutropenia or recurrent Grade 3 neutropenia in subsequent cycles.
Grade 3, absolute neutrophil count (ANC[b]) (<1000 to 500/mm^3) + Fever ≥38.5 ºC and/or infection	Discontinue IBRANCE until recovery to Grade ≤2<br>Continue with the next lower dose.
Grade 4	Suspend IBRANCE until recovery to Grade ≤2.<br>Continue with the next lower dose.
**Non-hematologic toxicities**	
Grade 1 or 2	No dose adjustment required.
Grade ≥3, non-hematologic toxicity (if it persists after medical treatment)	Discontinue until symptoms resolve to:<br>• Grade ≤1;

	• Grade ≤2 (if not considered a patient safety risk)<br>Continue with the next lower dose.

[a]The table applies to all hematologic adverse reactions except lymphopenia (unless associated with clinical events, such as opportunistic infections).
[b]ANC: Grade 1: ANC < LLN – 1500/mm^3; Grade 2: ANC 1000 - < 1500/mm^3; Grade 3: ANC 500 - < 1000/mm^3; Grade 4: ANC < 500/mm^3.

Table 1: Dose modifications

Dose modifications for use with potent CYP3A inhibitors

Concomitant use of potent CYP3A inhibitors (see section 5.5 below) should be avoided, and alternative concomitant medication with no or minimal CYP3A inhibition should be considered. If coadministration of IBRANCE with a potent CYP3A inhibitor cannot be avoided, reduce the dose of IBRANCE to 75 mg per day. When the potent inhibitor is discontinued, increase the dose of IBRANCE (after 3-5 inhibitor half-lives) to the dose used prior to initiation of the potent CYP3A inhibitor.

5.3 Treatment overdose

In the event of an overdose (defined as an administration greater than the dose specified in the protocol), investigators should contact the CRO assigned by the Sponsor, and additional monitoring of the patient's adverse events and laboratory abnormalities should be considered. Decisions regarding dose interruptions or modifications will be discussed by the investigator and the Sponsor's coordinating investigator based on the clinical evaluation of the patient. Information related to the amount of excess dose should be documented in the eCRF.

There is no known antidote for IBRANCE. Treatment of an overdose of IBRANCE should consist of general supportive measures.

5.4 Concomitant medication

All patients will be asked to provide a complete list of prescriptions and medications taken during the 4 weeks prior to the start of screening. Investigators should be informed, as soon as possible, of any medications taken from the start of screening until the first post-treatment follow-up visit.

All concomitant medications taken during the study will be recorded in the eCRF, including indication, dosing information, and dates of administration. If future changes are made to the list of permitted/prohibited medications, formal documentation will be provided and kept in the investigator's file. Such changes will be communicated to the research centers in the form of a letter.

Effect of CYP3A inhibitors

Coadministration of a potent CYP3A inhibitor (itraconazole) increased the plasma exposure of palbociclib in healthy subjects by 87%. Avoid concomitant use of CYP3A inhibitors (e.g., clarithromycin, indinavir, itraconazole, ketoconazole, lopinavir/ritonavir, nefazodone, nelfinavir, posaconazole, ritonavir, saquinavir, telaprevir, telithromycin, verapamil, and voriconazole). Grapefruit or grapefruit juice should be avoided during treatment with IBRANCE. If coadministration of IBRANCE with a potent CYP3A inhibitor cannot be avoided, reduce the dose of IBRANCE.

Effect of CYP3A inducers

Coadministration of a potent CYP3A inducer (rifampicin) reduced the plasma exposure of palbociclib in healthy patients by 85%. Avoid concomitant use of potent CYP3A inducers (e.g., phenytoin, rifampicin, carbamazepine, and St. John’s wort).

5.5 Pregnancy and lactation

Due to its mechanism of action, IBRANCE may cause fetal harm. Women of reproductive potential (and their partners) should use effective contraceptive measures throughout therapy with IBRANCE, and for at least 90 days after the last dose. Women should contact their physician if they become pregnant or if pregnancy is suspected during treatment with IBRANCE. Women should not breastfeed while being treated with IBRANCE because of the potential for serious adverse reactions in infants.

6. MEDICATION MANAGEMENT

6.1 Pharmaceutical form of palbociclib

IBRANCE 125 mg hard capsules: hard, opaque capsule with a caramel-colored body (with "PBC 125" printed in white) and a caramel-colored cap (with "Pfizer" printed in white). The capsule length is 21.7 ± 0.3 mm.

IBRANCE 100 mg hard capsules: hard, opaque capsule with a light orange body (with "PBC 100" printed in white) and a caramel-colored cap (with "Pfizer" printed in white). The capsule length is 19.4 ± 0.3 mm.

IBRANCE 75 mg hard capsules: hard, opaque capsule with a light orange body (with "PBC 75" printed in white) and a light orange cap (with "Pfizer" printed in white). The capsule length is 18.0 ± 0.3 mm.

6.2 Supply of study medication

Palbociclib (IBRANCE) is a drug developed and marketed by Pfizer. In the GEIS-51 trial, palbociclib will be supplied by Pfizer through the distribution company Alcura Health España, S.A.

6.3 Packaging, labelling and distribution

The contents of the investigational product label will meet the requirements of the applicable regulations in Spain. The study drug will be labelled and distributed directly to the pharmacy services of each participating center by the distribution company Alcura Health España, S.A., in coordination with the study CRO.

6.4 Storage in local pharmacies

Palbociclib should be stored in local pharmacies in a secure area under physical conditions appropriate for the product (palbociclib does not require special storage conditions). Access to and administration of the medication should be limited to authorized personnel of the center.

6.5 Dispensation to patients

The study drug should be dispensed by local pharmacies only to enrolled patients and according to protocol guidelines.

6.6 Drug accountability

In compliance with local regulatory requirements, designated site personnel should document the amount of medication received by the pharmacy from the Sponsor, the amount dispensed to trial patients, and the amount returned by patients to the pharmacy. Drug accountability documentation should be kept up to date throughout the course of the trial and will be filed according to the applicable regulations.

6.7 Treatment adherence

A record of the quantities of palbociclib dispensed and returned by each patient should be maintained and reconciled with the eCRF. During the course of the study and after its completion, all unused or expired trial medication will be accounted for and destroyed at the site.

6.8 Responsibilities of local pharmacies

The responsibilities of the designated responsible pharmacist at each participating site include, but are not limited to, ensuring that:

- The study drug is handled and stored safely and according to product-specific requirements;
- The study drug is dispensed only to trial patients according to protocol;
- There is a sufficient supply of the study drug for the treatment of patients, with timely notification to the Sponsor so that medication refills can be made;
- Study drug expiration dates are monitored, and that medication is used in order of expiration date (first to expire, first);
- Unused or expired medication is destroyed according to the local procedure at each site;
- Records of receipt, accounting and destruction of study medication are maintained.

7. PROCEDURES AND EVALUATIONS

7.1 Schedule of activities

Protocol section	Stages >>	PRE-TREAT.	TREATMENT												END TREAT.[2] (+/- 7 days)	FOLLOW-UP (+/- 7 days)
		Screening[1] (≤ 28 days from start of treatment)	C1 D1	C1 D14	C2 D1	C2 D14	C3 D1	C4 D1	C5 D1	C6 D1	C7 D1	C8 D1	C9 D1	C10 D1 … Cn D1		
7.2	**Patient information and consent**															
	Information to the patient	X (before screening)														
	Informed consent	X (before screening)														
7.3	**Eligibility and inclusion procedures**															
	CDK4 overexpression analysis	X														
	Eligibility confirmation	X														
	Patient recruitment	X														
7.4	**Clinical evaluations**															
	Demographics and medical history	X														
	ECOG	X	X	X	X	X	X	X	X	X	X	X	X	X	X	
7.5	**Laboratory tests[3] (+/- 3 days)**															
	Biochemistry[4]	X	X	X	X	X	X	X	X	X	X	X	X	X	X	
	Hematology[5]	X	X	X	X	X	X	X	X	X	X	X	X	X	X	
	Coagulation tests	X														
	Pregnancy test[6]	X						X			X			X[6]		
7.6	**Safety evaluations**															
	Physical examination[7]	X	X	X	X	X	X	X	X	X	X	X	X	X	X	
	Vital signs	X	X	X	X	X	X	X	X	X	X	X	X	X	X	
	Adverse events		Throughout the course of treatment													
	Concomitant medication	X	Throughout the course of treatment													
	ECG[8]	X														
	LVEF	X													X	
7.7	**Efficacy evaluations**															
	Radiological evaluation[9]	X	Every 8 weeks (irrespective of cycle delays)												X	
	Additional imaging tests[10]	X	Every 8 weeks													
	Central radiological review														X	
7.8	**Treatment control**															
	Medication dispensing [11]		X		X		X	X	X	X	X	X	X	X		
	Intake of medication		Palbociclib 125 mg once daily for 3 weeks followed by 1 week off (28-day cycles)													
	Treatment adherence		Every 4 weeks													
7.9	**Collection of biological samples**															
	Tumor block collection[12]	X														
	Blood collection[13]	X			X	Every 8 weeks (after each radiological evaluation)									X	
7.10	**Follow-up**															
	Follow-up visits															Every 3 months until death

Table 2: Schedule of activities

1. Documentation of *CDKN2A* mutation (chordoma cohort) and central diagnostic confirmation (STS, BS, and chordoma cohorts) is required for participation in the trial. For STS and BS cohort, a pre-screening test before screening is established, for the first CDK4 expression analysis done on already existing tumor block.
2. End-of-treatment evaluation must be done at the end of medication administration (within 4 weeks)
3. Baseline laboratory testing should be done within 7 days prior to initiation of treatment
4. Biochemistry: ALT, AST, total bilirubin, creatinine, alkaline phosphatase, glucose, albumin, total protein, LDH, chloride, phosphorus, sodium, potassium, calcium
5. Hematology: hematocrit, hemoglobin, leukocytes, red blood cells, neutrophils, platelets, RDW
6. Women of reproductive age. The pregnancy test should be done before starting treatment (within 7 days prior to inclusion) and then every 3 cycles until the end of treatment
7. In addition to other physical examinations (see section 7.6), weight and height are required at baseline, and height is not required on day 1 of each cycle
8. ECG within 14 days prior to enrollment
9. Preferably CT scan
10. FDG-PET, MRI diffusion, bone scan (if indicated)
11. Medication is dispensed to the patient on the first day of each cycle
12. A biopsy in the baseline period (within 28 days before the start of treatment) will be necessary if there is no archive tumor specimen obtained in the 3 months prior (no systemic treatment must be present in this period) to the initiation of treatment
13. Blood collections: (1) Within 72 hours pre-treatment, (2) at the end of the first cycle (day 28), (3) after each radiological evaluation

7.2 Patient information and consent

Pre-screening test (STS and BS cohort only)

An initial pre-screening test is established including a first expression analysis for CDK4 and p16. For this test, it is necessary that patients receive the patient information sheet (PIS) and sign the informed consent (IC) of the pre-screening stage.

Trial screening period

All candidate patients will receive the trial's own PIS, which describes, in simple language, the objectives, scope, procedures and relevant implications of the clinical trial. The PIS will include an IC form that must be signed by the patient (a prerequisite for participation in the study). Each patient must sign the written informed consent for the trial prior to the start of the screening process (before the protocol-specific assessments are carried out and before receiving the study treatment). The patient, if he or she agrees, must sign two separate consents: one for the main clinical trial and one for the collection of biological samples. Procedures that are part of routine practice (e.g., blood tests, imaging tests), performed before the consent is signed, can be used for screening purposes if these have been done as specified in the protocol.

7.3 Eligibility and inclusion procedures

CDK4 overexpression analysis (STS and BS cohort)

After the assignment of the pre-screening number, a first CDK4 overexpression analysis will be performed on an already existing tumor sample (obtained in a previous biopsy or surgery). If the result of the initial pre-screening test with the already existing sample is positive (CDK4 overexpression and low or normal expression of p16), the patient will have a new biopsy for CDK4 overexpression confirmation before entering the trial.

CDKN2A mutation verification (chordoma cohort)

In order for patients to participate in the trial, documentation confirming the CDKN2A mutation must be provided.

Biopsy for translational study

Each patient will have a new biopsy for the translational study prior to trial entry and within 28 days before the start of study treatment. The only exception to this new biopsy is if there is already a biopsy performed within 3 months prior to the start of study treatment, and the patient has not received any systemic treatment between that biopsy and the start of treatment.

The patient will not be able to participate in the study unless this tumor block, with adequate and sufficient material, is available.

Central confirmation of the diagnosis

Within the screening period, a central diagnostic confirmation will be performed on an existing tumor block (on file), at the Pathological Anatomy Service of Hospital Universitario Virgen del Rocío (Dr. David Marcilla).

Sending of samples and confirmation deadlines

Tumor samples will be sent by courier service to the central review laboratories, together with the anonymized origin pathology report corresponding to the samples. Central diagnostic confirmation will be available within approximately one week. Central confirmation of the diagnosis is mandatory in all cases without exception. Study treatment should not be started unless this confirmation is available.

A detailed manual for sample collection and shipment will be provided in the investigator’s file. The point of contact for coordinating tumor block shipments will be:

Sofpromed Investigación Clínica, SLU
Tel: +34 648 414 261
E-mail: ensayos@sofpromed.com

All tumor samples will be sent to:

CITIUS III - Manuel Losada Villasante Building
University of Seville
St. Doctor Rafael Martínez Domínguez s/n
41013 – Seville, Spain

Eligibility confirmation

The Principal Investigator (PI) of each center will be responsible for confirming that the patient to be recruited meets all the inclusion criteria and does not meet the exclusion criteria. The inclusion of the patient can only take place after this confirmation of eligibility.

Patient enrollment

After all screening procedures have been completed and candidate eligibility has been confirmed, patients will be enrolled in the study for initiation of treatment.

The procedure for enrolling a patient is described below:

- Complete and sign the patient enrollment form (the form must be signed by an authorized investigator)
- Submit (by fax or email) the enrollment form directly to the trial CRO:

Sofpromed Investigación Clínica, SLU
Fax: +34 971 570 222
E-mail: ensayos@sofpromed.com

- The trial CRO will perform the patient enrollment and send back (by email) to the center a notification confirming the inclusion of the patient. This notification will contain a unique patient number that will allow for the identification of the patient at all times during the trial.

A detailed manual on the patient enrollment procedure will be provided in the investigator’s file.

7.4 Clinical evaluations

Demographics and medical history

Patient demographics, past and present diseases and treatments, current medications, medications taken within 30 days prior to inclusion, date of diagnosis, previous antineoplastic therapies, and tumor staging will be collected. Any pre-existing toxicity (e.g., Fatigue grade 1) should be documented at this time.

ECOG

ECOG status will be assessed in the screening period. Then, it will be evaluated on days 1 and 14 of the first two cycles and on day 1 of the subsequent cycles. If patients complete treatment without disease progression (e.g., treatment withdrawal due to unacceptable toxicity), ECOG status assessments should be continued based on disease assessments until patients experience disease progression.

7.5 Laboratory tests

Laboratory tests must be performed as indicated in the schedule of activities. These tests can be done in the 7 days prior to the start of treatment to allow for flexibility. Tests could be performed more frequently if clinically indicated. The required tests are complete blood count and blood biochemistry at baseline or D1 and 14 in the first 2 cycles; from the 3rd cycle on D1. Coagulation tests only at baseline unless it is required more frequently. Pregnancy test at baseline and every 3 months thereafter.

All laboratory test values that are abnormal and clinically significant, during the period of the patient's participation in the study treatment and up to 28 days after the last dose, should be repeated until the values return to normal levels.

**Biochemistry**	
Renal function	Creatinine
Hepatic function	Albumin, alkaline phosphatase, AST (SGOT), ALT (SGOT) and total bilirubin
Electrolytes and others	Calcium, potassium, sodium, chloride, glucose, phosphorus and LDH
**Hematology**	Hematocrit, hemoglobin, leukocytes, red blood cells, neutrophils, platelets, RDW
**Coagulation tests**	Activated partial thromboplastin time (aPTT) and International Normalization Ratio (INR)
**Pregnancy test**	Palbociclib should not be administered to pregnant or breastfeeding women. For women of reproductive age, a negative blood or urine pregnancy test (minimum sensitivity 25 IU/L or β-HCG equivalent units) is required within 7 days prior to enrollment and every 3 cycles thereafter throughout the treatment.<br><br>A woman is eligible to enter and participate in the study if:<br><br>• She has no reproductive potential (e.g., physiologically unable to become pregnant), including women who have had a hysterectomy, a bilateral oophorectomy (ovariectomy), a bilateral tubal ligation or if she is postmenopausal.<br>• Female patients not using hormone replacement therapy (HRT) must have experienced cessation of menses for ≥ 1 year and must be over 45 years of age or, in questionable cases, must have a follicle-stimulating hormone value of >40 mIU/mL and an estradiol value of 40pg/mL (<140 pmol/L).<br>• Female patients using HRT must have experienced a cessation of menses for ≥ 1 year and must be over 45 years of age or must have had documented evidence of menopause based on follicle-

	stimulating hormone and estradiol concentrations prior to HRT initiation.<br><br>All the patients (men and women) must agree to use adequate contraceptive methods throughout treatment with palbociclib and for up to 90 days after completion of trial treatment. Acceptable contraceptive methods, which should be used consistently and according to product and physician instructions, are as follows:<br><br>• Oral contraceptive, either combined or progestogen alone<br>• Injectable progestogen<br>• Levonorgestrel implants<br>• Vaginal ring<br>• Percutaneous contraceptive patches<br>• Intrauterine device or intrauterine system with a documented error rate of less than 1% per year<br>• Sterilization of the male partner (vasectomy with documentation of azoospermia) prior to the woman's entry into the study, with this male being the patient's only partner.<br>• Double barrier method: condom and occlusive plug (cervical cap or diaphragm) with a spermicidal agent<br><br>Lactating patients should stop breastfeeding before the first dose of the investigational drug and should not breastfeed throughout treatment and for 14 days after the last dose.

Table 3: Laboratory tests

7.6 Safety evaluations

Any patient enrolled in the study who has received at least one dose of the trial medication will be evaluable for toxicity analysis. The safety profile will be characterized by treatment-emergent adverse events, vital signs, and laboratory abnormalities. Evaluation of adverse events will include type of event, incidence, severity (measured according to NCI-CTCAE 4.0 in the first phase of STS and bone sarcomas, and 5.0 in the second phase of chordomas), timing, and relationship to trial medication. Tumor-related signs and symptoms at baseline will be recorded as adverse events during the trial if they worsen in severity or increase in frequency. All adverse events will be recorded at each study visit.

Physical examination and vital signs

Pretreatment (screening): Evaluation by body system, height, weight, body surface area, and vital sign measurements (blood pressure and body temperature).

During treatment and follow-up: Evaluation by body system, weight, body surface area, and verification of blood pressure measurements.

Adverse events

CTC-AE version 4.0 will be used in the first phase (STS and bone sarcomas) and 5.0 in the second phase (chordomas) to evaluate the clinical safety of the treatment in the study. Patients' adverse events will be assessed at each clinic visit and as needed throughout the study.

Concomitant medication

Attempts should be made to avoid the use of any natural or herbal products, or other folk remedies. However, the use of these products, in addition to the use of vitamins or nutritional supplements and other concomitant medications, must be recorded in the eCRF. Any medication given in the study should be registered in the eCRF, specifying the name of the medication, dose, dates, and reasons for administration.

ECG

An electrocardiogram (ECG) should be obtained within 14 days prior to inclusion. Additional ECGs are not required at a later time if not clinically indicated.

LVEF

A MUGA or echocardiogram will be performed at baseline and at the end of treatment. The MUGA scanner is the preferred method for measurement of left ventricular ejection fraction (LVEF). If MUGA cannot be performed, an echocardiogram should be done. The same type of LVEF assessment should be performed at baseline and at the end of treatment.

7.7 Efficacy evaluations

CT scan

Imaging tests will preferably be performed with CT (contrast-enhanced, biphasic). Imaging methods will be used consistently throughout the course of the patient's evaluation in the study. The disease must be captured, including target and non-target lesions, at baseline. Baseline imaging tests should be completed within 28 days prior to the date of patient inclusion in the trial. Subsequently, until tumor progression, imaging studies to investigate the disease should be done every 8 weeks (every 2 cycles), regardless of cycle delays (to ensure a CT scan at 6 months (unless progression occurs earlier). Patients who have not progressed but have discontinued treatment for toxicity or other reasons (unrelated to tumor progression) will also be re-evaluated every 8 weeks unless they have started other anti-neoplastic therapy.

	**Choi**	**RECIST**
Complete response (CR)	Disappearance of all lesions.<br>No new lesions.	Disappearance of all lesions.
Partial response (PR)	Reduction in size ≥10% SLD or reduction in density ≥15% HU.<br>No new lesions.<br>Absence of unmeasurable disease progression.	Reduction of 30% of the total diameters of the main lesions.
Stable disease (SD)	CR, PR, or DP are not met.<br>No symptomatic deterioration attributable to tumor progression.	Between PR and DP.
Disease progression (DP)	Tumor growth ≥10% SLD and without PR criteria on radiological density in the CT scan.<br>New lesions. New intra-tumor nodules or growth of existing nodules or increase in the tissue portion of a hypodense lesion.	Increase of 20% of the total diameter or appearance of new lesions.

* SLD: Sum of the longest diameter (based on RECIST 1.1).

Table 4: Response evaluation criteria according to Choi/RECIST

To perform radiological studies, the following protocols must be applied:

Computed tomography (CT) scan

- Pre-contrast examination
- Contrast examination: It should be done using 120 ml of a conventional iodinated contrast agent, administered intravenously with an automated injector at a rate of 4 ml/s. Contrast examination will be done in: arterial phase (delay: bolus tracking), portal phase (60 s), delayed phase (6 min).
- Coronal multiplanar reconstruction (MPR) in the delayed phase.
- Tumor density will be determined by measuring the CT attenuation coefficient in Hounsfield Units (HU) by drawing a region of interest around the entire tumor margin, using a section thickness of 5 mm in the portal phase and in the delayed phase. Two-dimensional regions of interest will be drawn from the entire lesion, and all axial sections of the lesion will be included. The software will semiquantitatively calculate the mean tumor attenuation in HU, defined as the mean of all pixels included in the volume of interest.

- It is strongly recommended to use CT instead of NMRI.

Nuclear magnetic resonance imaging (NMRI)

- Morphological pre-contrast examination: always axial T1 SE, and T2 FSE (with or without fat pre-saturation) or STIR. Other sequences depending on local practice.
- Diffusion study: Axial b50, b400, b800, b1000.
- Dynamic examination:
- Before contrast injection: GE 3D TR less than 1 min variable angle (5, 10, 15, 20, 25, 28). During contrast injection: GE 3D TR less than 1 min (if possible 4 s) for 5 min.
- Morphological post-contrast examination: T1W coronal or sagittal sequence with fat saturation. Axial T1 SE without fat saturation.
- Post-contrast enhancement will be determined by measuring the MR signal, drawing a region of interest around the entire tumor margin in the subtracted series of Axial-T1-SE after CE minus Axial-T1-SE before contrast (parameters and the position must be identical in both sequences). The two-dimensional regions of interest of the entire lesion will be drawn, and all axial sections encompassing that lesion will be included. The software will semiquantitatively calculate the mean tumor signal, as the mean of all pixels included in the volume of interest.

Central review of radiological images

All radiological images will be reviewed centrally (at the national level) using a web-based platform. The person in charge of performing these reviews will be a radiologist designated by the Sponsor.

Upload of the images corresponding to the baseline period is required before the patient is recruited. If this is not feasible, they should be uploaded as soon as possible. All tumor imaging tests performed during the clinical trial should be uploaded to the imaging platform as soon as they become available. All images generated should be provided in DICOM format to allow for proper review.

All radiological studies will be anonymized before uploading, being identified only with the patient’s code in the trial.

For any issues regarding central imaging reviews, contact should be made with:

Sofpromed Investigación Clínica, SLU
Tel: +34 648 414 261
E-mail: ensayos@sofpromed.com

A detailed manual on image upload procedures will be provided in the investigator’s file.

7.8 Treatment control

Medication dispensing

Hospital pharmacies will dispense palbociclib capsules to patients sufficient to complete 4-week treatment periods.

Intake of medication

Study medication will be taken by patients as indicated in section 5 of this protocol (Treatment).

Adherence to treatment

In the patient’s intake diary, the patient will record the total number of palbociclib tablets taken. This information will be entered into the eCRF.

7.9 Collection of biological samples

Collection of tumor blocks

Tumor samples will be collected as outlined in section 12.3 of this protocol.

Collection of blood samples

Blood samples will be collected as indicated in section 12.3 of this protocol.

7.10 Follow-up

All patients should be followed until death, if possible. The date and cause of death must be documented on the eCRF. Follow-up visits should be done every 12 weeks (3 months) after tumor progression. These visits will assess the patient's status (alive, dead, lost), and note other possible antineoplastic treatments following trial treatment.

8. PHARMACOVIGILANCE

The ICH/GCP principles require that both investigators and sponsors follow specific procedures for reporting adverse events and adverse reactions in clinical trials. Any event involving adverse drug reactions, illnesses initiated during the study, or exacerbations of pre-existing conditions should be recorded. In addition, clinically significant changes in physical examination findings and abnormal test values (e.g., ECG) should also be recorded as adverse events. The criteria for determining whether a test result is objectively abnormal and should therefore be considered an adverse event are:

- The test result is associated with clinically significant symptoms, and/or
- The test result triggers a change in the dose of the study medication or a discontinuation, the need for additional treatment with concomitant medication or other therapy, and/or
- The test result triggers any of the events included in the definition of a serious adverse event, and/or
- The test result is considered an adverse event by the investigator.

8.1 Definitions

Adverse event (AE)

An AE is any untoward health incident that occurs to a trial subject to whom a drug has been administered, although it does not necessarily have a causal relationship with the drug.

Serious adverse event (SAE)

An SAE is any event detrimental to health which, at any dose, requires or prolongs hospitalization, results in permanent or significant disability or incapacity, leads to congenital anomaly or malformation, or is life-threatening or fatal.

Suspected Unexpected Serious Adverse Reaction (SUSAR)

A SUSAR is a serious adverse reaction whose nature, severity or outcome is not consistent with the referenced safety information.

8.2 Recording and reporting of adverse events

The investigator shall record and document any adverse events or abnormal laboratory results that the protocol deems critical to the safety evaluation and report them to the Sponsor in accordance with the reporting requirements and within the timeframes specified in the protocol, as indicated in article 41 of Regulation (EU) No. 536/2014 of the European Parliament and of the Council, of April 16, 2014.

The investigator shall report serious adverse events to the Sponsor without undue delay and within twenty-four hours of becoming aware of such events, unless, for certain serious adverse events, the protocol provides that immediate reporting is not required. The investigator, when appropriate, will send a follow-up report to the Sponsor to allow him or her to assess whether the serious adverse event has an impact on the benefit-risk ratio of the clinical trial. In the initial and follow-up reports, each trial subject will be identified only by his or her trial-specific subject identification code.

In the event that the death of a subject participating in a clinical trial has been reported, the investigator will provide the Sponsor and the RECm with all the additional information requested.

If the investigator becomes aware of a serious adverse event with a suspected causal relationship to the investigational medicinal product, which has occurred after the end of the clinical trial in a subject treated by the investigator, he or she will report this serious adverse event to the Sponsor without undue delay.

In any case, what is specified in the guidelines of the European Commission or, where appropriate, in the procedures established in the instructions for conducting clinical trials in Spain published by the Spanish Agency of Medicines and Medical Devices (AEMPS, by its Spanish acronym) must be considered.

AE registration

The Sponsor (through the assigned CRO in charge of pharmacovigilance) will collect the AEs up to 30 days after administering the last dose of the study drug. All adverse events will be recorded using medical terminology in the source document and in the electronic CRF. Investigators should assess the severity (grade) of the event using the NCI-CTCAE 4.0 (first phase; STS and bone sarcomas) and 5.0 (second phase; chordomas) criteria, assigning a possible relationship to the study drug and obtaining adequate information to determine the outcome and assess whether it is a serious adverse event requiring immediate reporting. The investigator should provide any information requested by the Sponsor in addition to that contained in the CRFs.

SAE reporting

Any serious adverse event (SAE) occurring from the moment the patient's informed consent is signed, during the clinical trial, and up to 30 days after receiving the last dose of the trial medication, whether drug-related or not, should be reported by the investigator. Moreover, any SAE occurring as a result of a protocol-specific process or intervention should also be reported. Beyond this time period, only those SAEs that are suspected to be related to the study drug shall be reported.

All SAEs suspected to be related to study treatment must be followed up after treatment/study withdrawal, until the event or its symptoms have been resolved or stabilized to a degree acceptable to the investigator and the Sponsor.

The local investigator team must notify the Sponsor (through the assigned CRO) of all female patient pregnancies occurring during the clinical trial within 24 hours of becoming aware of the event. The research staff of the center must also communicate the result of the pregnancy within 24 hours from the time they become aware of the situation.

The cause of death of a deceased clinical trial patient, whether it is an expected event or associated with the trial drug, is considered an SAE and, therefore, should be reported using the SAE form. The autopsy report must be sent to the Sponsor, indicating only the number of the patient in the trial.

Investigator teams must communicate by fax or email all SAEs and SUSARs within 24 hours to the trial CRO:

Sofpromed Investigación Clínica, SLU
Tel: +34 648 414 261
Fax: +34 971 570 222
E-mail: ensayos@sofpromed.com

The SAE report must include a complete summary, detailing the relevant aspects of the adverse event in question. When applicable, data from hospital reports or autopsies should be included. The follow-up reports of the event, up to event closure, should also be submitted to the CRO.

All SAEs related to the trial drug must be followed after study drug administration has been completed, until the event or its consequences have resolved or stabilized to a level acceptable to the investigator and the Sponsor.

8.3 Reporting of serious and unexpected adverse reactions

The Sponsor will notify the Spanish Agency of Medicines and Medical Devices of all suspected serious and unexpected adverse reactions associated with the investigational medicinal products of which he or she has become aware, which have occurred in the clinical trial, whether they occur in Spain or in other countries. Additionally, suspected serious and unexpected adverse reactions occurring outside the trial will be reported in accordance with the criteria established in the European Commission guidelines.

In all cases, said reporting will be done through the European database Eudravigilance_CTM.

The timeframe for notification of suspected serious and unexpected adverse reactions by the sponsor to the Spanish Agency of Medicines and Medical Devices will depend on the severity of the reaction and will be determined as follows:

a) In case of suspected serious and unexpected fatal or life-threatening adverse reactions, as soon as possible and, in any case, within seven days after the sponsor has become aware of the reaction.
b) In case of suspected serious unexpected adverse reactions that are not life-threatening, no later than fifteen days after the sponsor has become aware of the reaction.
c) In case of suspected serious and unexpected adverse reactions that are fatal or life-threatening and which were not initially considered as such, as soon as possible and in any case no later than seven days after the sponsor has become aware that the reaction is fatal or life-threatening.

Where necessary to ensure prompt notification, the sponsor may make an initial incomplete report that should be completed, to the extent possible, within the following eight days.

The reporting of serious and unexpected adverse reactions by the Sponsor to the Spanish Agency of Medicines and Medical Devices shall, in any case, comply with the criteria and procedure specified in articles 42, 45 and 46 of Regulation (EU) No. 536/2014 of the European Parliament and of the Council, of April 16, 2014.

8.4 Other obligations of the Sponsor in terms of safety

The Sponsor will keep a detailed record of all adverse events reported by the investigators. These adverse events will be submitted to the Spanish Agency of Medicines and Medical Devices upon request.

The Sponsor has the obligation to continuously evaluate the balance between the benefits and risks of the trial, which includes a continuous evaluation of the safety of the investigational medicinal products using all the information at his or her disposal. The Sponsor must also communicate without undue delay within fifteen calendar days to the Spanish Agency of Medicines and Medical Devices and to the RECm, by the means indicated in article 21, any information that significantly affects the benefit/risk ratio of the trial, with the exception of suspected serious and unexpected adverse reactions, which will be reported in accordance with article 51. Such communication will be made in accordance with the guidelines of the European Commission or, where appropriate, with the procedures established in the instructions for the conduct of clinical trials in Spain published by the Spanish Agency of Medicines and Medical Devices.

The Sponsor must promptly communicate to the investigators any important information that could negatively affect the safety of the subjects or the conduct of the trial. The communication of such information shall be concise and practical. The communication will conform to the criteria and procedure specified in the European Commission guidelines.

8.5 Safety reports

The Sponsor will prepare an annual safety report in which the safety of the investigational medicinal product is evaluated taking into consideration all available information. This report will be communicated to the Spanish Agency of Medicines and Medical Devices and to the RECm.

Independently of the annual safety report, the sponsor will prepare an “ad hoc” assessment report whenever there is a relevant safety issue. This report will be immediately submitted to the Spanish Agency of Medicines and Medical Devices and to the RECm as indicated in the previous section.

The submission of the annual safety report and other safety reports by the Sponsor shall in any case comply with the criteria and procedure specified in articles 43, 45 and 53 of Regulation (EU) No. 536/2014 of the European Parliament and of the Council, of April 16, 2014.

8.6 Surveillance of specific adverse reactions

Serious, life-threatening, or fatal ILD and/or pneumonitis may occur in patients treated with palbociclib. Patients should be monitored for pulmonary symptoms indicative of ILD/pneumonitis (e.g., hypoxia, cough, dyspnea). In patients with new or worsening respiratory symptoms suspected of having developed ILD/pneumonitis, treatment with palbociclib should be discontinued immediately and the patient should be evaluated. Palbociclib should be permanently discontinued in patients with severe ILD or pneumonitis.

9. DATA MANAGEMENT

9.1 Electronic case report form (eCRF)

In this protocol, the term electronic case report form (eCRF) refers to a web-based electronic data registry in which patient data will be collected.

Each participating center will be required to complete the eCRF for each patient enrolled. Only authorized and trained personnel may have access to enter and modify data in the eCRF. Individuals in charge of using the eCRF will receive a unique, non-transferable username and password.

The original electronic files of the eCRF are the sole property of the Sponsor and shall not be transferred in any manner to third parties, except for authorized use by representatives of the Sponsor or regulatory authorities, without the written permission of the Sponsor.

Investigators have the ultimate responsibility for the collection and communication of all clinical, safety and laboratory data entered into the eCRF, as well as other data forms and source documents, and must ensure that they are accurate, authentic, attributable, complete, consistent, legible, up-to-date, durable and available upon request.

The eCRF must be signed electronically by the investigator or by an authorized member of the research team, to attest that the data contained in the eCRF are true. Any change made to the data entered in the eCRF will be detected by a traceability and auditing system that will identify the old and new values of the data fields, the person who made the change, and the reason for the change (if necessary), thus not erasing the original data.

The data will be recorded in the eCRF following the GCP principles at the local recruiting center. The eCRF web application (designed according to FDA and EMA requirements) will remain stored in a secure server. All data processing steps, with the exception of data entry, will be performed centrally on a web server. All data will be stored in the central server. The server will be hosted by a professional provider contracted by the study CRO, ensuring effective security and backup mechanisms.

For data entry, the eCRF is fully based on a web interface. The data forms are displayed to users through their computer screens (PCs or laptops), in the form of HTML pages, using web browsers (IE 6+, FireFox 2+, Chrome, Safari 3+). No additional software is required to use the eCRF on the researcher's client computer (no plug-in installations or software or hardware adaptations are needed). The eCRF works independently of the computer's operating system (Windows/Mac).

The system will automatically check the validity and consistency of the data entered through edit checks and automatic queries. Erroneous or missing data can be corrected after interacting with the research teams. All corrections will be recorded and stored by the traceability mechanism integrated in the tool.

Apart from the investigator, only specifically authorized and trained people will be able to complete the eCRF. A system access control will be implemented based on an auditable log of user connections and disconnections. Automatic session disconnections will be scheduled after predefined periods of inactivity for security reasons. The eCRF will not display personally identifiable patient data (each patient will be identified with a unique code in the trial).

The study CRO will develop a comprehensive Data Management Plan, specifying the design of the eCRF/Database, as well as the procedures and policies related to data management.

9.2 Custody and maintenance of documentation

To enable evaluations, audits, and/or inspections by regulatory authorities or the Sponsor, the investigators agree to maintain records, including the identity of all participating patients (e.g., sufficient information to link the records), all original signed informed consent documents, safety norms, source documents, and appropriate documentation of relevant correspondence (e.g., letters, meeting minutes, phone call reports). Records must be kept by the investigator in accordance with the International Conference on Harmonization (ICH) and local regulations. In the event that the investigator for any reason is unable to continue with the trial records for the required period, the Sponsor must be notified. The study records must be transferred to a person in charge

designated by the Sponsor. Investigators should receive permission from the Sponsor before disposing of any records.

10. MONITORING

The study will be monitored by on-site visits (intermediate visits depending on the number of patients per site and closing visit) and supervised by telephone calls and period reviews of the eCRF, with sufficient frequency to ensure the following:

- That the investigational medicinal product is stored in adequate conditions, and that there is sufficient quantity of medication in each pharmacy for the trial patients.
- That the approved protocol and subsequent amended version, if any, are adhered to.
- That the investigators have received all documents and materials necessary to conduct the trial properly, in order to comply with applicable regulations.
- That the investigators and the staff of the research team have been adequately informed about the trial.
- That the trial data are complete and accurate (according to the monitoring plan):

  i. Informed consents (version, signature and date)
  ii. Eligibility criteria
  iii. Pre-inclusion tests (baseline)
  iv. Registry of adverse events
  v. Reporting of serious adverse events
  vi. Collection and storage of biological samples
  vii. Reconciliation of existing medication in pharmacies

- That the eCRFs are properly completed.
- That protocol deviations are reported according to GCP and applicable regulatory requirements.
- That the necessary actions are taken to avoid recurrence of the deviations detected.

The CRO assigned by the Sponsor will review the eCRF to verify compliance with the protocol and detect inconsistent or missing data. In the event of missing or anomalous data, queries will be sent to the centers involved for resolution. After data cleaning and necessary revisions, the data from the eCRF will be exported to the study database for further analysis.

The on-site monitoring visits will be carried out by the CRO assigned by the Sponsor. These visits will be made by qualified monitors, who must have access to the medical records after requesting permission from the researchers. Investigators should allow adequate time for these visits to take place, ensuring that the monitor has direct access to relevant documents.

When a visit is required, monitors will contact the center to arrange a date for the visit. Once the date has been confirmed, the center should ensure that the patient's relevant source documents are available for monitoring.

In the event that problems are detected during the course of the monitoring visit, the study CRO will collaborate with the Principal Investigator to resolve the issues and, if necessary, to determine the continued participation of the center in the study.

Monitors will review essential documents and conduct source data verification to confirm compliance with the protocol, and to ensure the protection of patients' rights as outlined in the Declaration of Helsinki 1964 (and subsequent amendments).

11. STUDY DESIGN AND STATISTICS

11.1 Study design

GEIS-51 is a multicenter, open-label, non-randomized, phase II clinical trial that initially had a single cohort of advanced sarcomas with CDK4 overexpression. Subsequently, a second phase (additional cohort) focused only on chordomas has been included. The study will be conducted only in Spain including the participation of third level hospitals.

11.2 Analytical variables

Main endpoint

- Efficacy measured by progression free survival (PFS) rate at 6 months, evaluated using RECIST 1.1. criteria.

Secondary endpoints

- Efficacy measured by overall response rate (ORR) (complete response [CR] and partial response [PR]), evaluated with RECIST 1.1. criteria. The evaluation criteria will be based on the identification of target lesions at baseline and their follow-up until tumor progression.
- Efficacy measured by response according to Choi criteria. The evaluation criteria will be based on the identification of target lesions at baseline and their follow-up until tumor progression.
- Efficacy measured by median PFS.
- Efficacy measured by PFS rate at 3 months.
- Overall survival (OS) measured from the date of initiation of treatment with palbociclib until the date of death, regardless of cause.
- Clinical benefit rate (CBR). Patients who have shown complete response, partial response or disease stabilization for 6 months or more, showing signs of clinical improvement, will be considered to have experienced clinical benefit.
- Safety profile of palbociclib, by evaluation of adverse events (type, incidence, severity, time of occurrence, related causes) observed on physical examinations and laboratory tests. Toxicity will be analyzed and tabulated using NCI-CTCAE 4.0 (first phase; STS and BS) and 5.0 (second phase; chordomas).

11.3 Sample size and statistical plan

Sample size – First phase (STS and BS)

According to the initial design for the first phase of STS and BS (Simon's two-stage), for an alpha error of 0.1 and a power (beta error) of 0.90, H0 14% (cut-off for active drugs, progression free survival rate at 6 months according to EORTC experience) and H1 35% of progression free survival rate at 6 months were estimated. In the first stage, 15 patients had to be recruited, of whom at least 3 had to have a progression free survival greater than 6 months. If this occurred, then a second stage would continue with further 23 treated patients, until reaching a total of 38 evaluable patients. If at least 9 patients had 6-month PFS or longer, further investigation of the drug would be warranted.

The design of the first phase has been amended preserving, to the extent possible, the differences between H0 and H1, due to low recruitment. The new approach is a Simon's two-stage Minimax design (type 1 and type 2 errors at 10%), H0 15% and H1 40%. If 2 or fewer successes are observed in the first 15 patients recruited, the trial will be stopped in this cohort with the conclusion that the investigational drug should not be further investigated in this subgroup of patients. In another case (3 or more successes in the first 15 patients recruited, PFS greater than 6 months) patient recruitment will continue up to 21 evaluable patients. If 6 or more successes are observed in these 21 patients, it will be concluded that the results of this trial deserve further investigation.

Sample size – Second phase (chordomas)

For the chordoma cohort (second phase), the primary variable of analysis is the rate of patients with PFS greater than 6 months. Since the mechanism of action is based on a reduction of viability in relation to G1 tumor cell arrest rather than apoptosis, it seems more reasonable to maintain this primary endpoint.

The first prospective study in chordoma with a systemic drug (9-nitro-camptothecin) showed a 33% PFS rate at 6 months[32]. Stacchiotti reported a 6-month PFS rate of 65%[33] while Schueltze obtained, with dasatinib, a 54% PFS rate at 6 months[34].

A Simon's two-stage Minimax design is considered, with type 1 and 2 errors of 10%. Success at 30% or less will be considered unacceptable and would not require further investigation (null hypothesis). Thus, the value of H0 will be 30%. Success in 60% of cases or more will be considered an acceptable result (H1, alternative hypothesis, 60%).

A total of 9 eligible and treated patients will be included in the first stage of this cohort. If 2 or fewer successes are seen, then the trial will be stopped in this cohort with the conclusion that the investigational drug should not be investigated further in chordoma. Otherwise (3 successes or more), recruitment will continue until 19 evaluable patients have entered the study. If 9 or more patients are successful among these 19 patients, it will be concluded that the results of this trial deserve further investigation.

Analytical methodology

For variables that follow binomial distributions (e.g., response rate), frequencies and percentages will be calculated, along with their exact (95%) confidence intervals. For time-to-event variables (e.g., PFS or OS), Kaplan-Meier estimates will be used. To analyze risk reduction and the influence of other variables on time-to-event variables, Cox Regression will be used. To correlate pharmacodynamic markers and biomarkers with clinical response, standard methods for bivariate and multivariate regression and correlation will be used. Multivariable methods will only be used if the required assumptions are verified.

11.4 Efficacy analysis

The populations for efficacy evaluations will be as follows:

- Intention-to-treat (ITT) population: Patients who signed the informed consent, having a central diagnosis and molecular expression confirmation, fulfilling all the inclusion criteria and none of the exclusion criteria.
- Per-protocol (PP) population: An efficacy analysis will be conducted in the PP population, which is defined as the subset of ITT population, without significant protocol deviations, with measurable disease (according to RECIST 1.1) at baseline, having had at least one cycle of study treatment and one radiological re-evaluation.

11.5 Safety analysis

Any patient included in the study who has received at least one dose of the investigational medication will be evaluable for the toxicity analysis. The safety profile will be characterized by adverse events related to study medication, vital signs, and laboratory test abnormalities. The evaluation of adverse events will include the type of event, severity (according to National Cancer Institute [NCI] Common Terminology Criteria for Adverse Events [CTCAE] grades, version 4.0 for the first phase –STS and bone sarcomas– and 5.0 for the second phase – chordomas), onset and end dates, the relationship and action taken with the study medication, other measures taken, and the outcome of the event. Tumor-related signs and symptoms observable in the pre-treatment (baseline) period will be recorded as adverse events during the trial if they worsen in severity or increase in frequency. In all visits, all adverse events will be recorded following the NCI-CTC version 4.0 terminology for the first phase (STS and bone sarcomas) and 5.0 for the second phase (chordomas).

12. TRANSLATIONAL SUBSTUDY

12.1 Rationale

New therapeutic schemes in sarcomas are urgently needed, mainly for patients diagnosed with advanced or metastatic disease and for whom there is no possibility of effective treatment. An important and attractive therapeutic strategy is based on cell cycle regulation. In fact, the mitotic index, together with the percentage of necrosis and differentiation, is important for the prognosis of the histological grade of sarcomas and the *cinsarc* genetic signature includes 67 genes involved in mitosis and chromosome integrity[6].

Cell division is an extremely well-regulated process and includes DNA replication and chromosome segregation into two independent cells, containing the same information. The transition between the different phases of the cell cycle occurs in an orderly manner and is regulated by numerous cellular proteins. The best-known key regulatory proteins are the serine (Ser/S)-threonine (Thr/T) cyclin-dependent protein kinases (CDKs), which are activated at specific stages of the cell cycle. These protein kinases remain stable during the cell cycle, but in turn the levels of their activator proteins, the cyclins, vary in order to specifically activate a particular CDK. Briefly, all three types of cyclin D (D1, D2, and D3) interacting with CDK4 and CDK6 are important for entering G1, and for regulating progression from G1 to S phase, cyclin E associates with CDK2. Furthermore, during S phase, cyclin A binds to CDK2 and to enter M phase the same cyclin complexes with CDK1. Lastly, progression through mitosis is regulated by cyclin B in association with CDK1. However, not only cyclin-CDK complexes are involved in the control of cell cycle progression. Indeed, several other protein kinases (e.g., Aurora kinases) and many transcription factors (e.g. p53) are associated with the regulation of cell division by distinct mechanisms.

Protein kinases CDK4 and CDK6 are an important part of the cell cycle. These kinases phosphorylate the tumor suppressor protein Rb (pRb), in response to mitogenic signals and/or anti-proliferative factors, allowing cell cycle progression and entry into G1[7]. Phosphorylation of Rb leads to its inactivation, which releases the transcription factor E2F, which is necessary for DNA synthesis and cell cycle progression. At this point, further phosphorylation of Rb is carried out by the cyclin E/CDK2 complex, which allows cell cycle progression through G1[8]. Also important is the non-catalytic function of cyclin D-CDK4/6 during G1, which isolate the Cip/Kip CDK inhibitors, $p21^{CIP1}$ and $p27^{KIP1}$, thus facilitating CDK2[9] activation and cell cycle progression. In addition, CDK inhibitors (CKIs) such as $p16^{INK4A}$ and $p27^{KIP1}$ develop an effective control of cell cycle progression, mainly by blocking the formation of cyclin-CDK complexes[10]. The G1-S phase transition is made according to various requirements by entering the cell into G0 or, under some circumstances, into senescence (permanent cell cycle arrest), in cases where the conditions necessary for cell cycle progression are not met. On the other hand, a typical characteristic of cancer is the deregulation of the cell cycle[1]. Several factors contribute to this, including the amplification or mutation of *CDK4* and *CDK6*, the amplification of cyclin-encoding genes, or the silencing of CKIs[2]. In effect, it is likely that many oncogenic processes depend on high CDK4 and CDK6 activity[35]. In sarcomas, CDK4 overexpression was detected in 92% of cases, in a series of 152 patients. In addition, high expression of proteins related to the Rb pathway is a common event in sarcomas[18]. These results suggest that CDK4 and CDK6 activity is a therapeutic target to be considered[27,36].

Palbociclib (PD0332991) is a selective CDK4/6 inhibitor with a half maximal inhibitory concentration (IC50) between 10 to 15nM in in vitro studies[22]. Palbociclib has been tested in several tumor types, including liposarcoma with CDK4 overexpression[24,25,37]. In a phase II trial in patients with well-differentiated or dedifferentiated liposarcoma, treatment with palbociclib demonstrated a favorable progression free survival (PFS) (PFS at 12 weeks of 57% and a median PFS of 17.9 weeks), in addition to a complete response[37]. In pre-clinical studies, the drug was shown to induce cell cycle arrest, senescence and cell death by apoptosis[28]. Cell cycle arrest in G1 is independent on Rb phosphorylation, suppressing tumor growth in in vivo models[26,38]. Nevertheless, mechanisms of resistance to CDK4 and CDK6 inhibitors have been described, such as loss of function of pRb[39] or $p16^{INK4A}$, or overexpression of cyclin D1 and CDK2[27]. Palbociclib also plays an important role in inhibiting FoxM1 phosphorylation[40–42]. CDK4/6 phosphorylates this transcription factor, stabilizing and activating FoxM1, which may lead to transcription of genes relevant to the G1-S transition, suppression of reactive oxygen species, protection of cells from senescence or transcription of pluripotency genes, which seem to play an important role in sarcomas (e.g. Sox2)[43–45]. Furthermore, FoxM1 is normally overexpressed in sarcomas, being negatively regulated by p53 and partially regulated by Rb and $p21^{CIP1}$. Thus, FoxM1 overexpression in sarcomas may be related to the frequent absence of p53 function observed in these tumors[44]. In fact, p53 mutations appear to be related to worse PFS and overall survival (OS) in patients with advanced STS, as recently described by our group[46]. Our group also demonstrated that PD0332991 induces senescence in in vitro and in vivo models of sarcoma. The response to palbociclib correlates with *CDK4* messenger RNA

(mRNA) levels, with drug showing activity in lines with high *CDK4* levels and low activity in lines with low *CDK4* expression and high p16^{INK4A} levels. Otherwise, palbociclib lowers tumor levels of CDK4[31].

Senescence is a process induced by different tumor suppressor proteins, such as p53, Rb, p21^{CIP1} and/or p16^{INK4A}, in response to an oncogenic stimulus. Currently, senescence is considered to play an important role in preventing the development of malignant lesions from pre-malignant cells[47]. The senescence process is usually associated with cell cycle arrest, but recently an important relationship with the anti-tumor immune response has been suggested[48]. In effect, several studies suggest that the destruction of senescent cells by cells of the immune system is a key step in preventing tumor development[49,50]. Immune cells involved in the immune surveillance of cancer include natural killer (NK) cells and macrophages[48].

Overall, palbociclib demonstrates activity in sarcomas, depending on CDK4 mRNA levels and in the context of p16^{ink4a} low or normal levels. Other proteins important in palbociclib activity are p53, p21^{CIP1}, p27^{KIP1}, Rb, FoxM1 and cyclin D1. In addition, the CDK4 inhibitor induces senescence, which is connected with the immune response. Nonetheless, the fact that palbociclib also inhibits CDK6, which is present in the immune system, may negatively affect the response. Thus, the effect of palbociclib at the level of immune response modulation will be a secondary objective of this study.

12.2 Objectives of the translational study

1.- Assess the expression of CDK4 (and p16^{ink4a}) mRNA and protein in tumor samples from patients diagnosed with sarcoma (blood and paraffin block), to identify the most reliable and effective method to select CDK4 and/or p16^{ink4a} as a predictive biomarker of the efficacy of palbociclib.

2.- Evaluate the predictive and/or prognostic value, at the mRNA and protein level, of p53, p21^{CIP1}, p27^{KIP1}, Rb, FoxM1 and cyclin D1 as potential biomarkers of the efficacy of palbociclib, in blood samples and in paraffin blocks (pre- and post-treatment).

3.- Analyze other senescence markers (combination of Ki67 negative with high density (>5 per nucleus) of γH2A.X) by immunohistochemistry of paraffin blocks (pre- and post-treatment). Pre- and post-treatment samples from each patient will be compared to determine senescence induced by palbociclib treatment.

4.- Correlate biomarkers that are predictive of palbociclib efficacy with clinical outcomes, such as PFS, OS and clinical benefit rates (complete response, partial response and stable disease).

5.- Evaluate the effect of palbociclib in modulating the immune response in paraffin block samples from patients undergoing chemotherapy. The evaluation will be performed on pre- and post-treatment samples from each patient.

12.3 Collection of biological samples

The samples to be collected in the study are described in the following table:

Nº	Type of sample	Contained in	Time of collection	Quantity	Enforceability	Purpose	Destination and person in charge
1	Tumor	Paraffin block	Already existing (biopsy or previous surgery)	1 block	Obligatory	First analysis of- CDK4 and p16 expression (pre-screening)	Instituto Biomedicina Sevilla – Dr. Amancio Carnero
2	Tumor	Paraffin block	Before enrollment	1 block	Obligatory	CDK4 and p16 expression confirmation	CITIUS III – Dr. David da Silva Moura
3	Tumor	Paraffin block	Already existing (biopsy or previous surgery)	1 block	Obligatory	Central confirmation of diagnosis	Hospital Universitario Virgen del Rocío – Dr. David Marcilla
4	Tumor	Paraffin block	Within 28 days prior to the start of treatment (if new) or within 3 months prior to the start of treatment (if already biopsied, in the absence of systemic treatment from collection to start of treatment)	1 block	Obligatory	Translational substudy	CITIUS III – Dr. David da Silva Moura
5	Tumor	Paraffin block	At the end of treatment with palbociclib	1 block	Optional		
6	Blood	Blood tube	Baseline (within 72h prior to initiation of palbociclib treatment)	25 ml	Obligatory		

7	Blood	Blood tube	Within 72h after completion of the first cycle of palbociclib (day 28)	25 ml	Obligatory		
8	Blood	Blood tube	After each radiological evaluation	25 ml	Obligatory		

Table 5: Biological samples of the trial

12.4 Methods and experiments

12.4.1 Protein evaluation

1) Building of tissue microarrays (TMA)

Four tru-cut biopsies (12G cylinders) will be compulsorily obtained at the time of diagnosis, which will be included in two paraffin blocks. In addition, post-treatment samples (two blocks) will be collected from a selective number of patients who accept the procedure. All cases will be reviewed by expert sarcoma pathologists. The blocks will be sent to the central translational laboratory, together with an anonymized copy of the pathology report. Hematoxylin-eosin (H&E) staining will be performed on each block in order to verify the existence of the tumor and thus select two representative areas.

Protein expression will be evaluated by immunohistochemical methods after the production of a tissue microarray (TMA) from pre- and post-treatment paraffin blocks. This method consists of carefully and simultaneously placing a high number of samples in a paraffin-embedded tissue. This allows the processing of a large number of samples under the same technical conditions. Whenever possible, two different tumor regions will be studied in each block. 3 µm sections will be obtained from each of the TMAs for immunohistochemical tests. Antibodies against the following proteins will be used: CDK4, p16^{ink4a}, p53, p21^{CIP1}, p27^{KIP1}, Rb, FoxM1, cyclin D1, Ki67 and γH2A.X. NK cell infiltrations will also be detected by immunohistochemistry with an anti-CD335 antibody (NKp46) and macrophages with an anti-CD68 antibody. A template will be prepared to ensure correct identification of each sample in the array.

2) Immunohistochemistry

Immunohistochemical studies will be performed on formalin-fixed, paraffin-embedded tissues, using 3-4 µm sections for this purpose in all cases. These studies will be done when inclusion of the tumor specimen in a TMA is not possible. The same antibodies described in the construction of tissue microarrays (TMA) will be used in the immunohistochemical studies.

Note: To improve the reliability of the immunohistochemical analysis, the results will be made by two pathologists. Additionally, complete block samples (one sample per 5 cases) will be reviewed in order to validate the results.

12.4.2 Transcriptional evaluation

1) RNA expression from paraffin-embedded tissue samples

RNA will be extracted and purified from paraffin blocks, and validation of individual genes will be performed by *qRT-PCR*. Total RNA will be isolated with the Recover All Total Nucleic Acid Isolation® kit (Ambion), according to the manufacturer's protocol, and 40 ng of total RNA will be used for reverse transcription using the High Capacity cDNA Reverse Transcription Kit (Applied Biosystems™ - Thermo Fischer Scientific), in the presence of MultiScribe™ reverse transcriptase and a random primer scheme to initiate cDNA synthesis. The cDNA obtained will be amplified and quantified by quantitative PCR using the TaqMan® Fast Advanced Master Mix Kit (Thermo Fischer Scientific). The relative expression of target genes will be normalized to β2-microglubin (*β2M*: (Hs99999907_m1, Applied Biosystems) and the expression in each sample will be compared to the control (RNA pool: Universal Human Reference RNA; Agilent Technologies). Individual quantification of gene expression will be performed by comparative CT (CT) method and relative expression will be calculated as 2-ΔCT. Genomic analysis includes the cyclin-dependent kinase *CDK4*, the cell cycle inhibitors *CDKN1A* (p21^{CIP1}), *CDKN2A* (p16^{ink4a}), *CDKN1B* (p27^{KIP1}), the tumor suppressor proteins *TP53* and *RB*, the cell cycle regulatory protein *CCND1* (cyclin D1), and the transcription factor *FOXM1*.

2) RNA expression from peripheral blood samples

Blood samples will be collected in EDTA tubes (2 x 10 ml per draw) at these times: (i) within 72 hours prior to initiation of palbociclib treatment, (ii) at the end of the first cycle (day 28) of palbociclib treatment, and (iii) after each radiological evaluation. After collection, blood samples will be stored at room temperature and sent within 24 hours to the central translational laboratory where the tubes will be stored at -20ºC or -80ºC, until use. RNA will be extracted from blood samples with the PAXgene RNA Kit Blood (PreAnalytiX). Reverse transcription, qRT-PCR and analysis of results will be carried out as described in point 1 of this section (Transcriptional evaluation). The same genes specified in point 1 of this section will be evaluated and the results obtained between pre- and post-treatment samples will be compared.

All samples from the translational substudy should be sent to the following address:

**CITIUS III - Manuel Losada Villasante building**<br>**University of Seville**<br>**Doctor Rafael Martínez Domínguez, no number**<br>**41013 – Seville, Spain**

For questions related to the shipment or collection of samples, please contact:

**Sofpromed Investigación Clínica, SLU**<br>**Tel: +34 648 414 261**<br>**E-mail: ensayos@sofpromed.com**

A manual for handling biological samples will be provided in the investigator's file.

13. ETHICAL AND REGULATORY ASPECTS

13.1 Ethics committee

The trial protocol and related documents will be submitted for review to the ethics committee, in accordance with the applicable regulations, prior to the start of the trial. Approval by the ethics committee is essential for initiation of the study.

13.2 Competent authority

The trial protocol and related documents should be submitted to the competent authority prior to the start of the clinical trial, as required by the Spanish Agency of Medicines and Medical Devices (AEMPS) of Spain. The approval of the AEMPS is indispensable for the initiation of the study.

13.3 Ethical framework of the study

This study will be conducted in compliance with the ethical principles originating from the Declaration of Helsinki adopted by the 18th World Medical Assembly, Helsinki, Finland, and its subsequent amendments. It will also be governed by the Good Clinical Practice (GCP) guidelines of the European Union (1990) Working Party on the Efficacy of Medicinal Substances (CPMP/ICH/135/95) and by the laws applicable in the country where the trial is being conducted.

13.4 Patient privacy

All parties involved in the trial must ensure the protection of patients' personal data and not include patients' names in any Sponsor forms, reports, publications, or in any other disclosures, except as required by law.

Patient names and other identifying data will be replaced by an alphanumeric code of the Sponsor.

In the event of data transfer, the Sponsor will maintain high standards of confidentiality and protection of patients' personal data.

To guarantee the confidentiality of the trial data in accordance with the Directive 95/46 of the European Parliament and 2001/20/EC, only the Sponsor or designated personnel for auditing or monitoring, the investigator and collaborators, the research ethics committees, or the one overseeing the site, and the relevant health authorities, will have access to the personal and clinical trial data.

The investigator should provide access to source documents and data for monitoring and audits.

The contents of the electronic case report forms (eCRFs), as well as the documents generated during the study, will be protected from unauthorized use by persons not involved in the trial, will be considered strictly confidential and will not be disclosed to third parties, except those specified in the preceding paragraphs.

13.5 Patient information sheet and informed consent

The patient information sheet (PIS) and informed consent (IC) must meet the requirements of the ICH and GCP guidelines, and local regulatory requirements. The PIS/IC in this study, and any changes made during its course, must be prospectively approved by the ethics committee prior to use. Hospital investigator teams should ensure that each patient, or appropriate representatives, are well informed about the nature and objectives of the study, including the potential risks associated with the trial. The investigators, or their designees, will obtain informed consents from each patient or the patient's legal representatives before any trial activity is started.

Patients should sign two separate informed consents: one for the overall clinical trial and one for the translational substudy with biological samples.

The patient should give his or her consent by signing the appropriate form in duplicate. For this purpose, each form should contain the signatures of both the investigator and the patient. The investigator shall retain a copy of the original of each signed consent form.

The patient should always give his or her written consent before being admitted to the study and before biological samples are taken.

13.6 Insurance policy

The Sponsor will take out a clinical trial insurance policy in accordance with applicable regulatory requirements. All patients in this study will be insured under this policy, which will be processed and funded by the Sponsor.

14. TRIAL GOVERNANCE AND RESPONSIBILITIES

14.1 Trial Steering Committee (TSC)

There will be a Trial Steering Committee (TSC) that will include the coordinating investigators of the study. The trial statistician will also be present at some TSC meetings and will provide the TSC with all information necessary for the analyses.

The responsibility of the TSC is to monitor and evaluate the progress of the trial and the safety of the participating subjects, and to ensure compliance with the protocol and the Good Clinical Practice principles.

The TSC will meet on a regular basis, in person at least once a year. A report of the findings and recommendations will be prepared after each meeting. This report will be sent to the Trial Management Group (TMG) and, if required, to relevant ethics committees and the competent authority.

14.2 Trial Management Group (TMG)

There will be a Trial Management Group (TMG) that will include the coordinating investigators and identified collaborators, including the trial statistician. Selected principal investigators will also be invited to join the TMG to ensure representation from a broad range of sites. The TMG will have operational responsibility in the conduct of the trial.

14.3 Responsibilities of the Sponsor

The Sponsor of the clinical trial is the Grupo Español de Investigación en Sarcomas (GEIS). GEIS will delegate to the CRO of the study a number of responsibilities that will be detailed in specified agreements. The Sponsor's responsibilities in the trial will also be specified in the contracts signed with each of the recruiting centers.

14.4 Responsibilities of the Principal Investigators

The responsibilities of each Principal Investigator and participating site will be detailed in a contract with the Sponsor.

The Principal Investigator's responsibilities include conducting the trial at his or her site in accordance with the trial protocol and applicable guidelines, local regulations, and GCP principles. These responsibilities include ensuring that:

- Applicable institutional and ethical approvals have been obtained before recruiting patients;
- Sufficient data are collected to allow for accurate matching between hospital records and eCRFs;
- Source data and all trial documents are accurate, complete and accessible for monitoring and audit visits;
- All personnel involved in the trial are trained and work according to applicable regulatory requirements;
- The original informed consent forms are personally signed and dated by both the patient and the investigator, and are kept in the investigator's file along with the patient information sheets provided to the patient;
- All essential documents are stored and maintained in accordance with local regulations;
- Trial protocol and guidelines are adhered to by trial personnel;
- The SAEs are communicated to the CRO of the study in the required timelines.

15. CONSIDERATIONS ON THE DEVELOPMENT OF THE STUDY

15.1 Inclusion of the study in clinical trial registries

The clinical trial will be registered in the *clinicaltrials.gov* database of the U.S. National Institute of Health, as well as in *clinicaltrialsregister.eu*.

15.2 Quality control

During the conduct of the study, the Sponsor, through his or her designated monitors, will conduct period monitoring visits to ensure that the protocol and good clinical practice (GCP) principles are being followed. The monitors will review source documents to verify that the data recorded in the eCRF are accurate. The investigators and participating sites will allow the Sponsor's monitors (or their subcontracted agents), as well as the competent authorities, to have direct access to the source documents to perform this verification.

Each participating center may be subject to reviews by the ethics committees, and/or quality control audits conducted by the Sponsor, or by companies that work with or on behalf of the Sponsor, as well as inspections carried out by the competent regulatory authorities.

Investigators and their staff must be available during monitoring visits and possible audits and/or inspections. Sufficient time should be dedicated to these processes.

15.3 Definition of end of study

The study will be considered closed from a regulatory point of view once the data related to the primary and secondary variables are sufficiently prepared for initial publication.

15.4 Criteria for suspension of the Sponsor

Premature termination of this study may occur due to the decision of a regulatory authority, a change of opinion by the ethics committees, medication safety issues, or by decision of the Sponsor. If the trial is terminated prematurely, the Sponsor will notify the investigators. After notification, each investigator must contact all participating patients and the hospital. All study materials should be collected and the eCRFs should be completed to the extent possible.

This trial may be terminated prematurely if, in the opinion of the Sponsor, there is reasonable and sufficient cause. In such a case, the researchers will receive written notice from the Sponsor explaining the termination of the study. Reasonable cause may include, but is not limited to:

- Observe unforeseen, considerable or unacceptable risks for the patients.
- Inability to include an acceptable number of patients.
- Insufficient compliance with protocol requirements.
- The existence of plans to modify, stop or terminate the development or production of the study medication.

15.5 Publication of results

The final publication of the trial results will be written by the coordinating investigators, based on the final analysis conducted.

The draft of the manuscript will be reviewed by all coordinating investigators and other co-authors. After review, the manuscript will be submitted to a high-impact scientific journal.

Regarding authorship, institutional inclusion of 5% of evaluable patients in the study will imply authorship (two names for 20% of inclusions, up to the number of authors allowed by the journal). Referring pathologists, radiologists and statisticians who have contributed to the trial will be included in the authors of the final manuscript.

All manuscripts will include an appropriate acknowledgements section, mentioning all investigators who have contributed to the trial, as well as other contributing parties.

The results of the translational substudy will be published in high-impact scientific journals, after publication of the main results of the clinical trial. The main authors will be the coordinators of the translational substudy. All centers that have contributed at least 5% of the material analyzed will be represented by a co-author. Centers that have contributed at least 15% of the analyzed material will be represented by two co-authors. These co-authors will be selected by each center internally (e.g., pathologist, molecular biologist, clinician). All centers that have provided material for analysis will be acknowledged.

APPENDIX A: CREATININE CLEARANCE (CrCl) MEASUREMENTS

Estimation of creatinine clearance using the Cockroft and Gault method:

$$\text{CrCl males (mL/min)} = \frac{[140 - \text{age (years)}] \times [\text{weight (kg)}]}{(72) \times [\text{Creatinine (mg/dL)}]}$$

$$\text{CrCl females (mL/min)} = \frac{(0.85) \times [140 - \text{age (years)}] \times [\text{weight (kg)}]}{(72) \times [\text{Creatinine (mg/dL)}]}$$

IS Units:

$$\text{CrCl males (mL/min)} = \frac{[140 - \text{age (years)}] \times [\text{weight (kg)}] \times (1{,}23)}{[\text{Creatinine } (\mu\text{mol/L})]}$$

$$\text{CrCl females (mL/min)} = \frac{[140 - \text{age (years)}] \times [\text{weight (kg)}] \times (1{,}05)}{[\text{Creatinine } (\mu\text{mol/L})]}$$

Calculation of creatinine clearance based on 24-hour urinary creatinine excretion and concurrent blood creatinine levels:

$\text{CrCl} = (Uc \cdot V) / C_{CR}$

Here, Uc is the urine creatinine concentration (mg/dL or μmol/L for SI units), V is the urine volume (in mL per minute of urine produced during the collection period), C_{CR} is the blood creatinine concentration (mg/dL or μmol/L for IS units), and CrCl is the creatinine clearance in mL per minute.

APPENDIX B: DECLARATION OF HELSINKI

Adopted by the
18th WMA General Assembly, Helsinki, Finland, June 1964
and amended by the
29th WMA General Assembly, Tokyo, Japan, October 1975
35th WMA General Assembly, Venice, Italy, October 1983
41st WMA General Assembly, Hong Kong, September 1989
48th WMA General Assembly, Somerset West, Republic of South Africa, October 1996
52nd WMA General Assembly, Edinburgh, Scotland, October 2000
53rd WMA General Assembly, Washington DC, USA, October 2002 (Note of Clarification added)
55th WMA General Assembly, Tokyo, Japan, October 2004 (Note of clarification added)
59th General Assembly, Seoul, Korea, October 2008
64th General Assembly, Fortaleza, Brazil, October 2013

Introduction

1.- The World Medical Association (WMA) has developed the Declaration of Helsinki as a statement of ethical principles for medical research involving human subjects, including research on identifiable human material and data.

The Declaration is intended to be read as a whole and each of its constituent paragraphs should be applied with consideration of all other relevant paragraphs.

2.- Consistent with the mandate of the WMA, the Declaration is addressed primarily to physicians. The WMA encourages others who are involved in medical research involving human subjects to adopt these principles.

General principles

3.- The Declaration of Geneva of the WMA binds the physician with the words "The health of my patient will be my first consideration," and the International Code of Medical Ethics declares that "A physician shall act in the patient's best interest when providing medical care."

4.- It is the duty of the physician to promote and safeguard the health, well-being and rights of patients, including those who are involved in medical research. The physician's knowledge and conscience are dedicated to the fulfilment of this duty.

5.- Medical progress is based on research that ultimately must include studies involving human subjects.

6.- The primary purpose of medical research involving human subjects is to understand the causes, development and effects of diseases and improve preventive, diagnostic and therapeutic interventions (methods, procedures and treatments). Even the best proven interventions must be evaluated continually through research for their safety, effectiveness, efficiency, accessibility and quality.

7.- Medical research is subject to ethical standards that promote and ensure respect for all human subjects and protect their health and rights.

8.- While the primary purpose of medical research is to generate new knowledge, this goal can never take precedence over the rights and interests of individual research subjects.

9.- It is the duty of physicians who are involved in medical research to protect the life, health, dignity, integrity, right to self-determination, privacy, and confidentiality of personal information of research subjects. The responsibility for the protection of research subjects must always rest with the physician or other health care professionals and never with the research subjects, even though they have given consent.

10.- Physicians must consider the ethical, legal and regulatory norms and standards for research involving human subjects in their own countries as well as applicable international norms and standards. No national or international ethical, legal or regulatory requirement should reduce or eliminate any of the protections for research subjects set forth in this Declaration.

11.- Medical research should be conducted in a manner that minimises possible harm to the environment.

12.- Medical research involving human subjects must be conducted only by individuals with the appropriate ethics and scientific education, training and qualifications. Research on patients or healthy volunteers requires the supervision of a competent and appropriately qualified physician or other health care professional.

13.- Groups that are underrepresented in medical research should be provided appropriate access to participation in research.

14.- Physicians who combine medical research with medical care should involve their patients in research only to the extent that this is justified by its potential preventive, diagnostic or therapeutic value and if the physician

has good reason to believe that participation in the research study will not adversely affect the health of the patients who serve as research subjects.
15.- Appropriate compensation and treatment for subjects who are harmed as a result of participating in research must be ensured.

Risks, burdens and benefits

16.- In medical practice and in medical research, most interventions involve risks and burdens.
Medical investigation involving human subjects may only be conducted if the importance of the objective outweighs the risks and burdens to the research subjects.
17.- All medical research involving human subjects must be preceded by careful assessment of predictable risks and burdens to the individuals and groups involved in the research in comparison with foreseeable benefits to them and to other individuals or groups affected by the condition under investigation.
Measures to minimise the risks must be implemented. The risks must be continuously monitored, assessed and documented by the researcher.
18.- Physicians may not be involved in a research study involving human subjects unless they are confident that the risks have been adequately assessed and satisfactorily managed.
When the risks are found to outweigh the potential benefits or when there is conclusive proof of definitive outcomes, physicians must assess whether to continue, modify or immediately stop the study.

Vulnerable groups and individuals

19.- Some groups and individuals are particularly vulnerable and may have an increased likelihood of being wronged or of incurring additional harm.
All vulnerable groups and individuals should receive specifically considered protection.
20.- Medical research with a vulnerable group is only justified if the research is responsive to the health needs or priorities of this group and the research cannot be carried out in a non-vulnerable group. In addition, this group should stand to benefit from the knowledge, practices or interventions that result from the research.

Scientific requirements and research protocols

21.- Medical research involving human subjects must conform to generally accepted scientific principles, be based on a thorough knowledge of the scientific literature, other relevant sources of information, and adequate laboratory and, as appropriate, animal experimentation. The welfare of animals used for research must be respected.
22.- The design and performance of each research study involving human subjects must be clearly described and justified in a research protocol.
The protocol should contain a statement of the ethical considerations involved and should indicate how the principles in this Declaration have been addressed. The protocol should include information regarding funding, sponsors, institutional affiliations, potential conflicts of interest, incentives for subjects and information regarding provisions for treating and/or compensating subjects who are harmed as a consequence of participation in the research study.
In clinical trials, the protocol must also describe appropriate arrangements for post-trial provisions.

Research ethics committees

23.- The research protocol must be submitted for consideration, comment, guidance and approval to the concerned research ethics committee before the study begins. This committee must be transparent in its functioning, must be independent of the researcher, the sponsor and any other undue influence and must be duly qualified. It must take into consideration the laws and regulations of the country or countries in which the research is to be performed as well as applicable international norms and standards but these must not be allowed to reduce or eliminate any of the protections for research subjects set forth in this Declaration.
The committee must have the right to monitor ongoing studies. The researcher must provide monitoring information to the committee, especially information about any serious adverse events. No amendment to the protocol may be made without consideration and approval by the committee. After the end of the study, the researchers must submit a final report to the committee containing a summary of the study’s findings and conclusions.

Privacy and confidentiality

24.- Every precaution must be taken to protect the privacy of research subjects and the confidentiality of their personal information.

Informed consent

25.- Participation by individuals capable of giving informed consent as subjects in medical research must be voluntary. Although it may be appropriate to consult family members or community leaders, no individual capable of giving informed consent may be enrolled in a research study unless he or she freely agrees.

26.- In medical research involving human subjects capable of giving informed consent, each potential subject must be adequately informed of the aims, methods, sources of funding, any possible conflicts of interest, institutional affiliations of the researcher, the anticipated benefits and potential risks of the study and the discomfort it may entail, post-study provisions and any other relevant aspects of the study. The potential subject must be informed of the right to refuse to participate in the study or to withdraw consent to participate at any time without reprisal. Special attention should be given to the specific information needs of individual potential subjects as well as to the methods used to deliver the information.

After ensuring that the potential subject has understood the information, the physician or another appropriately qualified individual must then seek the potential subject's freely-given informed consent, preferably in writing. If the consent cannot be expressed in writing, the non-written consent must be formally documented and witnessed.

All medical research subjects should be given the option of being informed about the general outcome and results of the study.

27.- When seeking informed consent for participation in a research study the physician must be particularly cautious if the potential subject is in a dependent relationship with the physician or may consent under duress. In such situations the informed consent must be sought by an appropriately qualified individual who is completely independent of this relationship.

28.- For a potential research subject who is incapable of giving informed consent, the physician must seek informed consent from the legally authorised representative. These individuals must not be included in a research study that has no likelihood of benefit for them unless it is intended to promote the health of the group represented by the potential subject, the research cannot instead be performed with persons capable of providing informed consent, and the research entails only minimal risk and minimal burden.

29.- When a potential research subject who is deemed incapable of giving informed consent is able to give assent to decisions about participation in research, the physician must seek that assent in addition to the consent of the legally authorised representative. The potential subject's dissent should be respected.

30.- Research involving subjects who are physically or mentally incapable of giving consent, for example, unconscious patients, may be done only if the physical or mental condition that prevents giving informed consent is a necessary characteristic of the research group. In such circumstances the physician must seek informed consent from the legally authorised representative. If no such representative is available and if the research cannot be delayed, the study may proceed without informed consent provided that the specific reasons for involving subjects with a condition that renders them unable to give informed consent have been stated in the research protocol and the study has been approved by a research ethics committee. Consent to remain in the research must be obtained as soon as possible from the subject or a legally authorised representative.

31.- The physician must fully inform the patient which aspects of their care are related to the research. The refusal of a patient to participate in a study or the patient's decision to withdraw from the study must never adversely affect the patient-physician relationship.

32.- For medical research using identifiable human material or data, such as research on material or data contained in biobanks or similar repositories, physicians must seek informed consent for its collection, storage and/or reuse. There may be exceptional situations where consent would be impossible or impracticable to obtain for such research. In such situations the research may be done only after consideration and approval of a research ethics committee.

Use of placebo

33.- The benefits, risks, burdens and effectiveness of a new intervention must be tested against those of the best proven intervention(s), except in the following circumstances:

Where no proven intervention exists, the use of placebo, or no intervention, is acceptable; or where for compelling and scientifically sound methodological reasons the use of any intervention less effective than the best proven one, the use of placebo, or no intervention is necessary to determine the efficacy or safety of an

intervention and the patients who receive any intervention less effective than the best proven one, placebo, or no intervention will not be subject to additional risks of serious or irreversible harm as a result of not receiving the best proven intervention.

Extreme care must be taken to avoid abuse of this option.

Post-trial provisions

34.- In advance of a clinical trial, sponsors, researchers and host country governments should make provisions for post-trial access for all participants who still need an intervention identified as beneficial in the trial. This information must also be disclosed to participants during the informed consent process.

Research registration and publication and dissemination of results

35.- Every research study involving human subjects must be registered in a publicly accessible database before recruitment of the first subject.
36.- Researchers, authors, sponsors, editors and publishers all have ethical obligations with regard to the publication and dissemination of the results of research. Researchers have a duty to make publicly available the results of their research on human subjects and are accountable for the completeness and accuracy of their reports. All parties should adhere to accepted guidelines for ethical reporting. Negative and inconclusive as well as positive results must be published or otherwise made publicly available. Sources of funding, institutional affiliations and conflicts of interest must be declared in the publication. Reports of research not in accordance with the principles of this Declaration should not be accepted for publication.

Unproven interventions in clinical practice

37.- In the treatment of an individual patient, where proven interventions do not exist or other known interventions have been ineffective, the physician, after seeking expert advice, with informed consent from the patient or a legally authorised representative, may use an unproven intervention if in the physician’s judgement it offers hope of saving life, re-establishing health or alleviating suffering. This intervention should subsequently be made the object of research, designed to evaluate its safety and efficacy. In all cases, new information must be recorded and, where appropriate, made publicly available.

REFERENCES

1. Billingsley, K. G. *et al.* Pulmonary metastases from soft tissue sarcoma: analysis of patterns of diseases and postmetastasis survival. *Ann Surg* **229**, 602–612 (1999).

2. Reichardt, R., Tilgner, J., Hohenberger, P. & Dörken, B. Dose-intensive chemotherapy with ifosfamide, epirubicin, and filgrastim for adult patients with metastatic or locally advanced soft tissue sarcoma: A phase II study. *Journal of Clinical Oncology* **16**, (1998).

3. le Cesne, A. *et al.* Randomized phase III study comparing conventional-dose doxorubicin plus ifosfamide versus high-dose doxorubicin plus ifosfamide plus recombinant human granulocyte-macrophage colony-stimulating factor in advanced soft tissue sarcomas: A trial of the European Organization for Research and Treatment of Cancer/Soft Tissue and Bone Sarcoma Group. *J Clin Oncol* **18**, 2676–2684 (2000).

4. Santoro, A. Advanced soft tissue sarcoma: how many more trials with anthracyclines and ifosfamide? *Ann Oncol* **10**, 151–154 (1999).

5. van Glabbeke, M. *et al.* Prognostic factors for the outcome of chemotherapy in advanced soft tissue sarcoma: an analysis of 2,185 patients treated with anthracycline-containing first-line regimens--a European Organization for Research and Treatment of Cancer Soft Tissue and Bone Sarcoma Group Study. *J Clin Oncol* **17**, 150–157 (1999).

6. Chibon, F. *et al.* Validated prediction of clinical outcome in sarcomas and multiple types of cancer on the basis of a gene expression signature related to genome complexity. *Nat Med* **16**, 781–787 (2010).

7. Malumbres, M. & Barbacid, M. Cell cycle, CDKs and cancer: a changing paradigm. *Nat Rev Cancer* **9**, 153–166 (2009).

8. Sherr, C. Cell cycle control and cancer. *Harvey Lect* **96**, 73–92 (2000).

9. Sherr, C. J. & Roberts, J. M. CDK inhibitors: positive and negative regulators of G1-phase progression. *Genes Dev* **13**, 1501–1512 (1999).

10. Carnero, A. & Hannon, G. J. The INK4 family of CDK inhibitors. *Curr Top Microbiol Immunol* **227**, 43–56 (1998).

11. Malumbres, M. & Barbacid, M. Cell cycle kinases in cancer. *Curr Opin Genet Dev* **17**, 60–65 (2007).

12. Hanahan, D. & Weinberg, R. A. Hallmarks of cancer: the next generation. *Cell* **144**, 646–674 (2011).

13. Gil, J. & Peters, G. Regulation of the INK4b-ARF-INK4a tumour suppressor locus: all for one or one for all. *Nat Rev Mol Cell Biol* **7**, 667–677 (2006).

14. Puyol, M. *et al.* A synthetic lethal interaction between K-Ras oncogenes and Cdk4 unveils a therapeutic strategy for non-small cell lung carcinoma. *Cancer Cell* **18**, 63–73 (2010).

15. Drosten, M. *et al.* Genetic analysis of Ras signalling pathways in cell proliferation, migration and survival. *EMBO J* **29**, 1091–1104 (2010).

16. Santamaría, D. *et al.* Cdk1 is sufficient to drive the mammalian cell cycle. *Nature 2007 448:7155* **448**, 811–815 (2007).

17. Knudsen, E. S. & Wang, J. Y. J. Targeting the RB-pathway in cancer therapy. *Clin Cancer Res* **16**, 1094–1099 (2010).

18. Sabah, M., Cummins, R., Leader, M. & Kay, E. Aberrant expression of the Rb pathway proteins in soft tissue sarcomas. *Appl Immunohistochem Mol Morphol* **14**, 397–403 (2006).

19. Zhao, R., Choi, B. Y., Lee, M. H., Bode, A. M. & Dong, Z. Implications of Genetic and Epigenetic Alterations of CDKN2A (p16(INK4a)) in Cancer. *EBioMedicine* **8**, 30–39 (2016).

20. Liu, T. *et al.* CDK4 expression in chordoma: A potential therapeutic target. *J Orthop Res* **36**, 1581–1589 (2018).

21. von Witzleben, A. *et al.* Preclinical Characterization of Novel Chordoma Cell Systems and Their Targeting by Pharmocological Inhibitors of the CDK4/6 Cell-Cycle Pathway. *Cancer Res* **75**, 3823–3831 (2015).

22. Logan, J. E., Mostofizaden, N., Desai, A. J., von Euw, E. & Conklin, D. PD-0332991, a potent and selective inhibitor of cyclin-dependent kinase 4/6, demonstrates inhibition of proliferation in renal cell carcinoma at nanomolar concentrations and molecular markers predict for sensitivity. *Anticancer Res* **33**, 2997–3004 (2013).

23. Finn, R. S. *et al.* PD 0332991, a selective cyclin D kinase 4/6 inhibitor, preferentially inhibits proliferation of luminal estrogen receptor-positive human breast cancer cell lines in vitro. *Breast Cancer Res* **11**, (2009).

24. Zhang, Y. X. *et al.* Antiproliferative effects of CDK4/6 inhibition in CDK4-amplified human liposarcoma in vitro and in vivo. *Mol Cancer Ther* **13**, 2184–2193 (2014).

25. Dickson, M. A. *et al.* Phase II trial of the CDK4 inhibitor PD0332991 in patients with advanced CDK4-amplified well-differentiated or dedifferentiated liposarcoma. *J Clin Oncol* **31**, 2024–2028 (2013).

26. Wiedemeyer, W. R. *et al.* Pattern of retinoblastoma pathway inactivation dictates response to CDK4/6 inhibition in GBM. *Proc Natl Acad Sci U S A* **107**, 11501–11506 (2010).

27. Dickson, M. A. Molecular pathways: CDK4 inhibitors for cancer therapy. *Clin Cancer Res* **20**, 3379–3383 (2014).

28. Eilers, G. *et al.* CDKN2A/p16 Loss Implicates CDK4 as a Therapeutic Target in Imatinib-Resistant Dermatofibrosarcoma Protuberans. *Mol Cancer Ther* **14**, 1346–1353 (2015).

29. Malumbres, M. & Barbacid, M. Is Cyclin D1-CDK4 kinase a bona fide cancer target? *Cancer Cell* **9**, 2–4 (2006).

30. Collins, I. & Garrett, M. D. Targeting the cell division cycle in cancer: CDK and cell cycle checkpoint kinase inhibitors. *Curr Opin Pharmacol* **5**, 366–373 (2005).

31. Perez, M., Muñoz-Galván, S., Jiménez-García, M. P., Marín, J. J. & Carnero, A. Efficacy of CDK4 inhibition against sarcomas depends on their levels of CDK4 and p16ink4 mRNA. *Oncotarget* **6**, 40557–40574 (2015).

32. Scimeca, P. G., James-Herry, A. G., Black, K. S., Kahn, E. & Weinblatt, M. E. Chemotherapeutic treatment of malignant chordoma in children. *J Pediatr Hematol Oncol* **18**, 237–240 (1996).

33. Stacchiotti, S. *et al.* Phase II study of imatinib in advanced chordoma. *J Clin Oncol* **30**, 914–920 (2012).

34. Schuetze, S. M. *et al.* Phase 2 study of dasatinib in patients with alveolar soft part sarcoma, chondrosarcoma, chordoma, epithelioid sarcoma, or solitary fibrous tumor. *Cancer* **123**, 90–97 (2017).

35. O'Leary, B., Finn, R. S. & Turner, N. C. Treating cancer with selective CDK4/6 inhibitors. *Nat Rev Clin Oncol* **13**, 417–430 (2016).

36. Tadesse, S., Yu, M., Kumarasiri, M., Le, B. T. & Wang, S. Targeting CDK6 in cancer: State of the art and new insights. *Cell Cycle* **14**, 3220–3230 (2015).

37. Dickson, M. A. *et al.* Progression-Free Survival Among Patients With Well-Differentiated or Dedifferentiated Liposarcoma Treated With CDK4 Inhibitor Palbociclib: A Phase 2 Clinical Trial. *JAMA Oncol* **2**, 937–940 (2016).

38. Vlenterie, M. *et al.* Targeting Cyclin-Dependent Kinases in Synovial Sarcoma: Palbociclib as a Potential Treatment for Synovial Sarcoma Patients. *Ann Surg Oncol* **23**, 2745–2752 (2016).

39. Malorni, L. *et al.* A gene expression signature of retinoblastoma loss-of-function is a predictive biomarker of resistance to palbociclib in breast cancer cell lines and is prognostic in patients with ER positive early breast cancer. *Oncotarget* **7**, 68012–68022 (2016).

40. Castellano, D. E. *et al.* RB-independent activity of Cdk4/6 in bladder cancer. *https://doi.org/10.1200/JCO.2016.34.15_suppl.e16011* **34**, e16011–e16011 (2016).

41. Euw, E. M. von *et al.* Abstract 1321: Identification of markers of sensitivity and resistance to palbociclib (PD0332991) in melanoma. *Cancer Res* **74**, 1321–1321 (2014).

42. Acevedo, M. *et al.* A CDK4/6-Dependent Epigenetic Mechanism Protects Cancer Cells from PML-induced Senescence. *Cancer Res* **76**, 3252–3264 (2016).

43. Anders, L. *et al.* A systematic screen for CDK4/6 substrates links FOXM1 phosphorylation to senescence suppression in cancer cells. *Cancer Cell* **20**, 620–634 (2011).

44. Kelleher, F. C. & O'Sullivan, H. FOXM1 in sarcoma: role in cell cycle, pluripotency genes and stem cell pathways. *Oncotarget* **7**, 42792–42804 (2016).

45. Riggi, N. *et al.* EWS-FLI-1 modulates miRNA145 and SOX2 expression to initiate mesenchymal stem cell reprogramming toward Ewing sarcoma cancer stem cells. *Genes Dev* **24**, 916–932 (2010).

46. Martin-Broto, J. *et al.* Randomized Phase II Study of Trabectedin and Doxorubicin Compared With Doxorubicin Alone as First-Line Treatment in Patients With Advanced Soft Tissue Sarcomas: A Spanish Group for Research on Sarcoma Study. *J Clin Oncol* **34**, 2294–2302 (2016).

47. Dimri, G. P. What has senescence got to do with cancer? *Cancer Cell* **7**, 505–512 (2005).

48. Iannello, A. & Raulet, D. H. Immunosurveillance of senescent cancer cells by natural killer cells. *Oncoimmunology* **3**, (2014).

49. Kang, T. W. *et al.* Senescence surveillance of pre-malignant hepatocytes limits liver cancer development. *Nature 2011 479:7374* **479**, 547–551 (2011).

50. Xue, W. *et al.* Senescence and tumour clearance is triggered by p53 restoration in murine liver carcinomas. *Nature* **445**, 656–660 (2007).
